# The Cytotoxic Activity of Secondary Metabolites from Marine-Derived *Penicillium* spp.: A Review (2018–2024)

**DOI:** 10.3390/md23050197

**Published:** 2025-04-30

**Authors:** Shuncun Zhang, Huannan Wang, Chunmei Sai, Yan Wang, Zhongbin Cheng, Zhen Zhang

**Affiliations:** 1 School of Pharmacy, Binzhou Medical University, 346 Guanhai Road, Yantai 264003, China; 18397016309@163.com; 2 School of Pharmacy, Jining Medical University, 669 Xueyuan Road, Rizhao 276800, China; wanghuannan0322@126.com (H.W.); saichunmei1980@163.com (C.S.); 18264955901@163.com (Y.W.); 3 Key Laboratory of Tropical Biological Resources of Ministry of Education, School of Pharmaceutical Sciences, Hainan University, Haikou 570228, China

**Keywords:** marine fungi, *Penicillium*, secondary metabolites, cytotoxic activity

## Abstract

Marine-derived *Penicillium* spp., including *Penicillium citrinum*, *Penicillium chrysogenum*, and *Penicillium sclerotiorum*, have emerged as prolific producers of structurally diverse secondary metabolites with cytotoxic activity. This review systematically categorizes 177 bioactive compounds isolated from marine *Penicillium* spp. between 2018 and 2024, derived from diverse marine environments such as sediments, animals, plants, and mangroves. These compounds, classified into polyketides, alkaloids, terpenoids, and steroids, exhibit a wide range of cytotoxic activities. Their potency is categorized as potent (<1 μM or <0.5 μg/mL), notable (1–10 μM or 0.5–5 μg/mL), moderate (10–30 μM or 5–15 μg/mL), mild (30–50 μM or 15–25 μg/mL), and negligible (>50 μM or >25 μg/mL). The current review highlights the promising role of marine *Penicillium* spp. as a rich repository for the discovery of anticancer agents and the advancement of marine-inspired drug development.

## 1. Introduction

Cancer remains a prominent global health challenge, with 2022 surveillance data from the International Agency for Research on Cancer (IARC) revealing 20 million incident cases (including non-melanoma skin cancers) and 9.7 million cancer-attributed deaths [1]. The lifetime cancer risk approaches 20%, with gender-specific mortality rates of 11.1% in males and 8.3% in females. Lung carcinoma leads in global incidence (2.5 million cases, 12.4%), followed sequentially by breast (11.6%), colorectal (9.6%), prostate (7.3%), and gastric malignancies (4.9%). Gender-specific analysis identifies breast and lung cancers as principal determinants of female and male oncologic morbidity/mortality, respectively [1,2]. Notably, sustained mortality reductions in developed nations (e.g., 2.3% annual decline in the US) reflect enhanced early detection protocols and precision oncology innovations in targeted therapies and immunomodulation [3].

Modern oncology employs multimodal therapeutic strategies, including surgical resection, radiation therapy, systemic chemotherapy, molecularly targeted agents, immune checkpoint inhibitors, endocrine therapies, and investigational approaches [4,5]. Despite its systemic efficacy, conventional chemotherapy faces substantial clinical limitations due to dose-limiting toxicities and acquired resistance mechanisms, representing a persistent therapeutic challenge in cancer management. Tumors constitute complex ecosystems comprising transformed cells, stromal components, and remodeled extracellular matrices. The tumor microenvironment (TME) orchestrates critical oncogenic processes through dynamic interactions between malignant cells and resident stromal populations, including tumor-associated macrophages, cancer-associated fibroblasts, endothelial cells, and pericytes. These interactions drive tumor initiation, progression, and metastatic dissemination through paracrine signaling and extracellular matrix remodeling [6]. Chemoresistance emerges through multifactorial mechanisms, including drug metabolism alterations, target modification, efflux transporter upregulation, enhanced DNA repair capacity, and apoptotic pathway dysregulation [4]. Recent evidence highlights the context-dependent nature of resistance mechanisms, which are influenced by the cellular phenotype, tumor evolution dynamics, and cumulative mutational load [5].

Natural products exert multimodal anticancer effects through the simultaneous modulation of molecular targets and oncogenic pathways, including apoptosis regulation, proliferation inhibition, migration suppression, angiogenesis blockade, and metastatic prevention. These bioactive compounds initiate intracellular signaling cascades culminating in cancer cell death, offering promising therapeutic potential against drug-resistant malignancies [7]. While terrestrial sources have historically dominated in anticancer drug discovery, marine ecosystems provide unparalleled chemical diversity due to their unique ecological pressures and evolutionary adaptations [8,9].

Marine-derived bioactive compounds exhibit multi-target anticancer efficacy through cross-pathway modulation. Accumulated evidence confirms that the NF-κB, mTOR, and PI3K/Akt pathways constitute primary therapeutic targets of marine-origin antitumor agents [10]. Emerging pharmacological insights further reveal the coordinated regulation of tumorigenic processes through MAPK cascades, Wnt/β-catenin signaling, and their intricate network crosstalk. The MAPK superfamily, encompassing the ERK, JNK, and p38 subfamilies, serves as a master regulator of cellular homeostasis, governing proliferation, differentiation, and apoptotic machinery [11]. In the canonical ERK pathway, Ras GTPase activation initiates membrane recruitment of RAF kinases, which phosphorylate MEK1/2 to subsequently activate ERK1/2 via dual phosphorylation at conserved Thr/Tyr motifs (T202/Y204 in ERK1; T183/Y185 in ERK2). Phosphorylated ERK1/2 dimers translocate to the nucleus, where they mediate transcriptional activation through the kinase-dependent modification of effector proteins (Elk-1, c-Myc, CREB), thereby promoting cell cycle progression (via cyclin D1 induction), apoptosis resistance (through Bad inactivation), and metastatic potential [12]. p38 MAPK signaling, a stress-responsive hub, requires MKK3/6-mediated phosphorylation at T180/Y182 for activation. Nuclear-localized p38 orchestrates inflammatory responses (TNF-α/IL-1β production), cell cycle arrest (p21-dependent), and mitochondrial apoptosis (Bax/Bak activation) via the phosphorylation of transcription factors (ATF2, MEF2C) and chromatin remodelers (MSK1/2). Contemporary studies highlight its non-canonical roles in post-transcriptional regulation through RNA-binding protein phosphorylation (e.g., HuR-mediated mRNA stabilization), autophagosome biogenesis, and stress granule assembly [13]. JNK signaling (c-Jun N-terminal kinase), an evolutionarily conserved stress-activated pathway, demonstrates synergistic interactions with the NF-κB and JAK/STAT networks to balance survival–autophagy–apoptosis triage. Mechanistically, JNK activation induces cytoprotective autophagy through Bcl-2 phosphorylation, representing an adaptive mechanism against chemotherapy-induced apoptosis [14]. The canonical Wnt/β-catenin axis, frequently targeted by marine compounds, requires specific ligand–receptor engagement (e.g., Wnt3a/10b) with Frizzled receptors and LRP5/6 co-receptors [15]. This interaction inhibits β-catenin degradation, allowing nuclear accumulation and TCF/LEF-dependent transcription of oncogenic targets—a process validated in multiple marine compound studies addressing epithelial-derived malignancies [15,16].

Marine ecosystems, encompassing microbial communities (e.g., bacteria, fungi), macroalgae, seagrasses, and diverse fauna (including corals, ascidians, and crustaceans), have been established as treasure troves of structurally unique secondary metabolites. These compounds exhibit potent cytotoxicity against a broad spectrum of cancer cell lines, positioning marine-derived bioactive agents as innovative chemical scaffolds for therapeutic development and pivotal resources for next-generation anticancer drug discovery [17,18]. The clinical validation of marine-derived pharmaceuticals is underscored by 17 FDA/EMA-approved agents as of 2022 [19]. Notably, four structurally distinct marine-inspired compounds—lurbinectedin, polatuzumab vedotin, enfortumab vedotin, and belantamab mafodotin—have recently gained regulatory approval for treating ovarian cancer, diffuse large B-cell lymphoma, urothelial carcinoma, and multiple myeloma, respectively [18]. Furthermore, cytarabine (Cytosar-U^®^) represents a paradigm-shifting achievement. This sponge-derived nucleoside analog selectively targets *S*-phase progression through DNA polymerase inhibition, demonstrating efficacy against acute leukemias and meningeal malignancies. Similarly, ziconotide (Prialt^®^), a *Conus magus* venom peptide, revolutionized pain management through selective *N*-type calcium channel blockade, providing an opioid alternative for refractory cancer- and AIDS-related pain [20]. Contemporary marine drug discovery continues to yield structurally novel leads, exemplified by the tubulin-binding polyketides PM050489 and PM060184 currently in clinical development [21,22,23]. Among marine-derived producers, fungal secondary metabolites have emerged as a prolific source of anticancer scaffolds, with *Penicillium* spp. contributing 22% of characterized marine fungal natural products. This genus represents a chemically diverse reservoir of structurally unique compounds with novel mechanisms of action, underscoring its significance in marine natural product research [24,25,26,27].

The marine-derived didemnin family has emerged as a novel anticancer pharmacophore with multi-target therapeutic potential, demonstrating synergistic antitumor mechanisms through molecular-level interventions. Current research confirms their ability to (1) disrupt ribosomal function to suppress global protein biosynthesis, (2) concurrently activate mitochondrial-mediated and death receptor-mediated apoptosis pathways, (3) induce cell cycle checkpoint dysregulation in malignant proliferation, (4) remodel tumor microenvironment immunogenicity, and (5) exhibit inhibitory effects against viral-associated oncogenesis [28]. Comparative pharmacological evaluations revealed that didemnin B demonstrates superior cytotoxic activity, which facilitated its progression to clinical trials. However, phase II studies identified dose-limiting toxicities including neuromuscular impairment and hepatotoxicity, prompting structural optimization efforts [29]. The semi-synthetic derivative plitidepsin (Aplidin^®^), featuring a dehydrodidemnin B scaffold, exemplifies successful structure–activity relationship optimization. Xenograft models demonstrate an enhanced therapeutic index compared to the parental compound through improved pharmacokinetic profiles and reduced off-target distribution [30]. Clinical validation comes from the NAPOLY-1 trial, where plitidepsin achieved a 20.7% overall response rate in relapsed/refractory multiple myeloma, with manageable hematological toxicity [30]. Notably, combination regimens demonstrate translational potential. Synergistic effects were observed when didemnin B was combined with sorafenib in lymphoma models [31]. A phase Ib trial (NCT02371135) demonstrated a 60% disease control rate in patients with T-cell lymphoma receiving plitidepsin–gemcitabine–carboplatin regimen without overlapping toxicity profiles [31,32].

## 2. Secondary Metabolites of Marine-Derived *Penicillium* spp.

The seminal discovery of penicillin from *Penicillium* spp. by Alexander Fleming in 1928 marked the dawn of modern mycology-based drug discovery. Over the past three decades (1991–2023), marine-derived *Penicillium* strains have emerged as prolific producers of structurally diverse secondary metabolites with significant pharmacological potential. Extensive investigations have characterized their unique biosynthetic pathways, while mechanistic studies have elucidated structure–activity relationships underlying their anti-inflammatory and anticancer properties [33,34,35,36,37,38]. Marine ecosystems harbor phylogenetically diverse *Penicillium* spp. with exceptional ecological plasticity, rendering this ascomycete genus a strategic resource for cytotoxic lead discovery. Systematic bioprospecting spanning 1991–2017 yielded more than 200 cytotoxic metabolites with antitumor potential from marine-derived *Penicillium* spp. [33,39]. This comprehensive review systematically evaluates cytotoxic and antitumor secondary metabolites from marine-derived *Penicillium* spp. reported between 2018 and 2024, encompassing 177 characterized compounds with 93 structural novelties (52.5% discovery rate). The identified metabolites are classified according to their biosynthetic origins, with particular emphasis on their molecular mechanisms of action and structure–activity relationship (SAR) profiles. In this paper, the cytotoxicity of related compounds was evaluated by the IC_50_ value to assess its strength, and the specific efficacy levels were potent (<1 μM or <0.5 μg/mL), notable (1–10 μM or 0.5–5 μg/mL), moderate (10–30 μM or 5–15 μg/mL), mild (30–50 μM or 15–25 μg/mL), and negligible (>50 μM or >25 μg/mL) [33].

**Figure 1:**
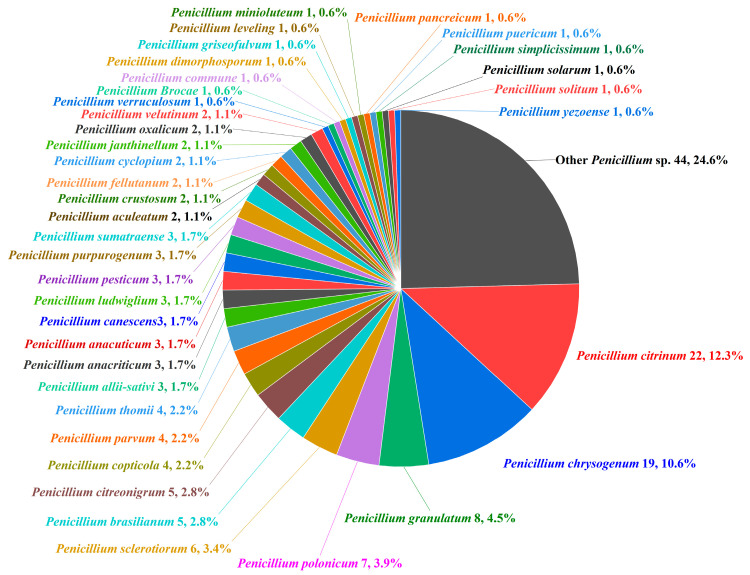
Distribution of selected cytotoxic compounds from marine-derived *Penicillium* spp.

**Figure 2:**
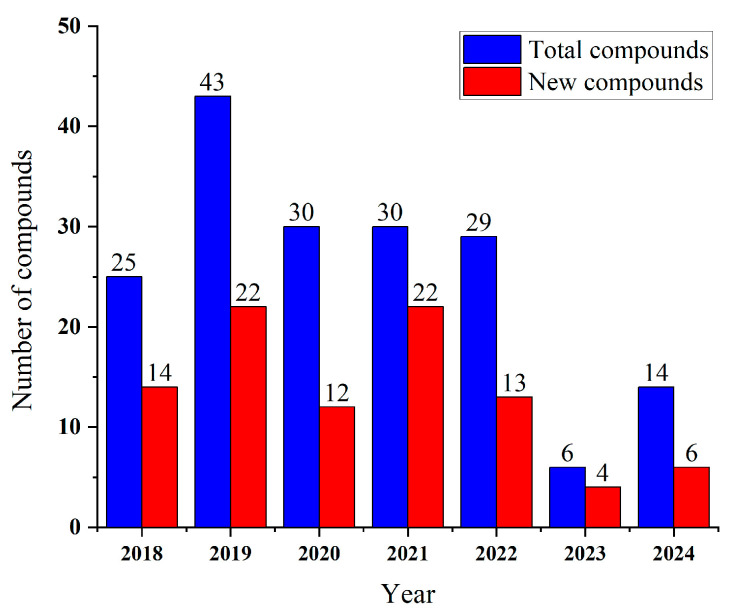
Annual statistics of *Penicillium*-derived cytotoxic compounds.

*Penicillium* spp. have emerged as prolific producers of bioactive secondary metabolites, with *Penicillium citrinum* (22, 12.3%), *Penicillium chrysogenum* (19, 10.6%), *Penicillium granulatum* (8, 4.5%), and *Penicillium polonicum* (7, 3.9%) constituting the major sources of anticancer compounds, collectively accounting for 31.3% of the characterized molecules. Additional significant contributors include *Penicillium sclerotiorum* (6, 3.4%), *Penicillium brasilianum* (5, 2.8%), and *Penicillium citreonigrum* (5, 2.8%), while 31 other species each yield 0.6–2.2% of metabolites, including *Penicillium copticola*, *Penicillium parvum*, and *Penicillium thomii* (4, 2.2% each). Notably, 24.6% of compounds are derived from unidentified *Penicillium* strains (Figure 1). Recent investigations of cytotoxic compounds derived from marine *Penicillium* spp. have revealed remarkable chemical diversity, with approximately 52.5% being newly identified metabolites. The annual statistics presented in Figure 2 demonstrate this sustained discovery trend, detailing both cumulative bioactive compounds and novel cytotoxic agents identified through annual screening, thereby establishing marine *Penicillium* as a continuously valuable resource for drug discovery research [40,41,42].

**Figure 3:**
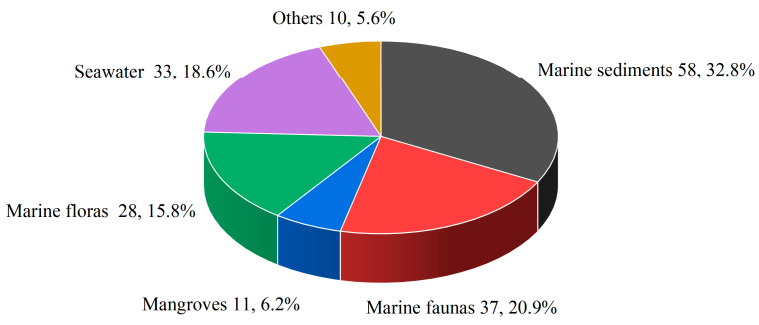
Sources of selected cytotoxic compounds from marine-derived *Penicillium* spp.

As prolific producers of bioactive secondary metabolites, marine-derived *Penicillium* spp. demonstrate ubiquitous colonization across pelagic ecosystems, inhabiting marine sediments, sea water, and epibiotic substrates. This ecological versatility correlates with exceptional biosynthetic plasticity through niche-specific adaptation, manifested as specialized metabolic arrays fulfilling distinct ecological functions. In this review, a statistical analysis of marine *Penicillium* spp. habitats revealed distinct ecological preferences: Marine sediments (32.8%), characterized by high organic content and microbial diversity, served as the predominant source. Symbiotic associations with marine faunas (20.9%) provided specialized ecological niches, while marine floras (15.8%), particularly algae and seagrasses, offered nutrient-rich substrates. Pelagic environments contributed 18.6% of isolates, with mangroves (6.2%) representing unique terrestrial–marine interfaces supporting fungal growth through complex ecological interactions. Notably, 5.6% of isolates originated from atypical environments (wastewater, sand, soil), demonstrating the genus’s remarkable ecological plasticity and adaptive capacity across diverse habitats (Figure 3).

**Figure 4:**
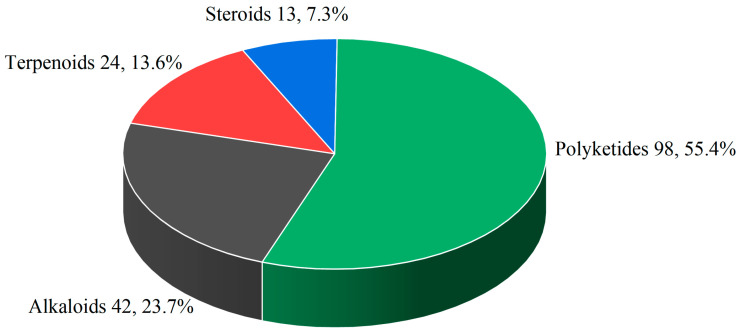
Chemical class distribution of selected marine-derived *Penicillium* secondary metabolites.

**Figure 5:**
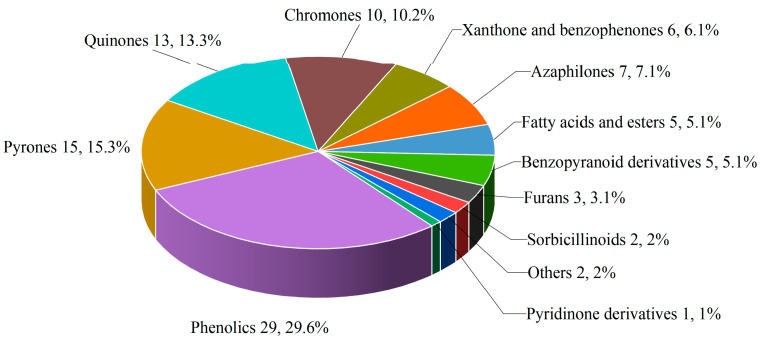
Chemical class distribution of selected polyketides produced by *Penicillium* spp.

Marine-sourced *Penicillium* spp. exhibit extraordinary chemodiversity through their biosynthetic repertoire, generating structurally distinct specialized metabolites spanning alkaloids, polyketides, terpenes, steroids, and other types. The 177 characterized compounds predominantly comprise polyketides (98, 55.4%), followed by alkaloids (42, 23.7%), terpenoids (24, 13.6%), and steroids (13, 7.3%) (Figure 4). Structural diversity analysis identified 23 distinct subclasses: polyketides encompass 12 subfamilies (Figure 5), including azaphilones, chromones, xanthones, and quinones; alkaloids are represented by 7 structural classes, such as indole derivatives and pyrrolizidines; terpenoids consist of 2 subgroups (sesquiterpenoids, meroterpenoids and diterpenoids); and steroids.

## 3. Chemical Diversity of Marine-Derived *Penicillium* Secondary Metabolites

### 3.1. Polyketides

**Figure 6:**
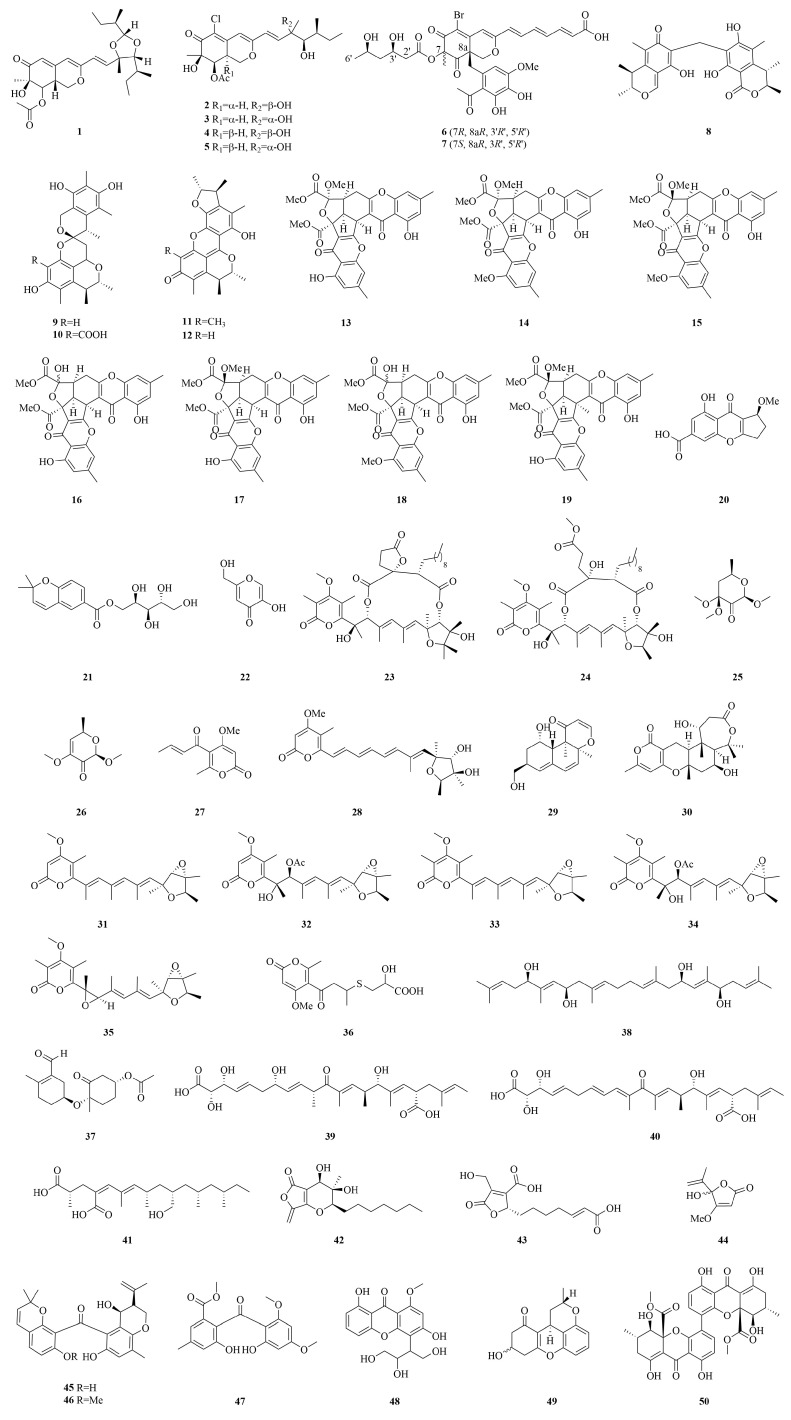
The chemical structures of cytotoxic polyketides (**1**–**50**).

**Figure 7:**
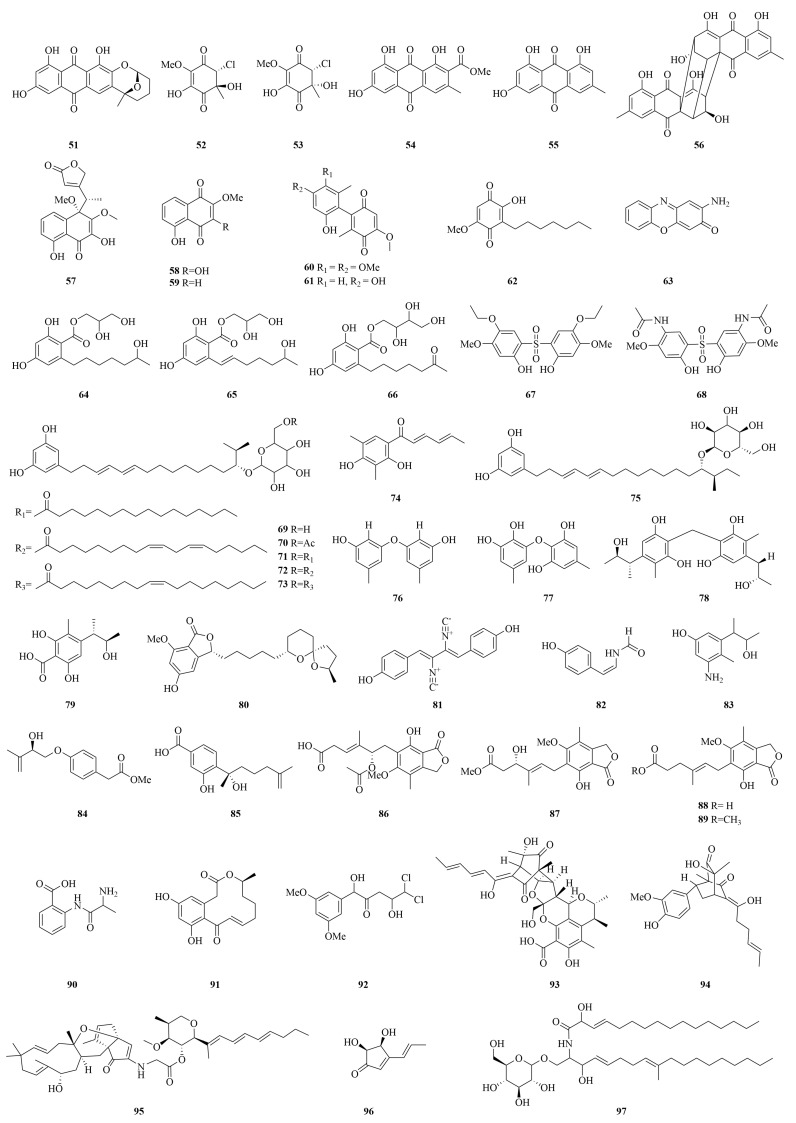
The chemical structures of cytotoxic polyketides (**51**–**97**).

Polyketides, biosynthesized through the polyketide synthase (PKS)-mediated sequential condensation of carboxylic acid precursors, represent the most structurally diverse class of fungal secondary metabolites. These compounds exhibit remarkable chemical variability and broad-spectrum bioactivities, accounting for 55.4% of cytotoxic compounds identified from marine-derived *Penicillium* spp. This predominance underscores their significance in marine natural product-based drug discovery [43,44]. The structural diversity of anticancer polyketides derived from marine *Penicillium* spp. is shown in Figure 6 and Figure 7.

#### 3.1.1. Azaphilones/Azaphilonoids

Azaphilones (or azaphilonoids) represent a structurally diverse class of fungal polyketide-derived molecules, characterized by a highly oxygenated pyranoquinone bicyclic core. To date, 13 distinct structural subtypes have been characterized within this class of compounds [45]. Recent investigations of marine-derived *Penicillium sclerotiorum* E23Y-1A yielded a novel compound with a dioxolane-based framework, penidioxolane C (**1**), which demonstrated selective cytotoxicity against five human tumor models—chronic myelogenous leukemia (K562), hepatocarcinoma (BEL-7402/HepG2), gastric adenocarcinoma (SGC-7901), non-small-cell lung carcinoma (A549), and cervical carcinoma (HeLa)—with IC_50_ values ranging from 23.94 to 60.66 µM [46].

The marine alga-associated *Penicillium sclerotiorum* KM265451.1 yielded two novel azaphilones—8a-epi-hypocrellone A (**2**) and 8a-epi-eupenicilazaphilone C (**3**)—alongside known analogs **4** and **5**. Compound **2** demonstrated selective neuroblastoma cytotoxicity against SH-SY5Y cells with an IC_50_ value of 35.6 µM. A comparative SAR analysis of **2**–**5** revealed α-H (R_1_) and β-hydroxyl (R_2_) substituents as critical determinants for target selectivity, suggesting that spatial orientation influences bioactivity [47].

Bromophilones A (**6**) and B (**7**), representing the first reported azafluorones featuring a methylene-bridged phenyl-pyranoquinone scaffold, were isolated from *Penicillium canescens* associated with the Mediterranean sponge *Agelas oroides*. Stereochemical configuration significantly influenced bioactivity, with **7** demonstrating notable cytotoxicity against L5178Y murine lymphoma (IC_50_, 8.9 µM) and A2780 human ovarian carcinoma (IC_50_, 2.7 µM) cells, while its stereoisomer **6** exhibited markedly reduced potency [48].

#### 3.1.2. Benzopyranoid Derivatives

Marine-derived benzopyranoids exhibit unparalleled structural diversity in their pharmacophoric architectures, highlighting their potential as privileged scaffolds for pharmaceutical innovation. The analysis of marine fungal isolates (2000–2023) reveals that 33.9% of characterized benzopyran derivatives exhibit antitumor potential, underscoring their privileged scaffold status in drug discovery [49]. The starfish-sourced *Penicillium* sp. GGF16-1-2 was found to produce carbon-bridged dicitrinone G (**8**). This architecturally distinct dimer demonstrated moderate cytotoxicity against pancreatic adenocarcinoma cell lines BXPC-3 and PANC-1, with IC_50_ values of 12.25 and 24.33 μM, respectively. Notably, it exhibited 1.5- to 2.0-fold enhanced potency compared to doxorubicin hydrochloride (18.24 μM for BXPC-3 and 24.00 μM for PANC-1). Furthermore, a mechanistic study suggested that compound **8** may promote apoptosis in BXPC-3 cells by influencing the activation of CASP3 [50]. Concurrently, a chemical investigation of sponge-associated *Penicillium citrinum* SCSIO41017 yielded citrinin derivatives **9**–**12**, with the tetracyclic meroterpenoid **12** demonstrating marked antineoplastic activity against estrogen receptor-positive breast adenocarcinoma MCF-7 cells, with an IC_50_ value of 1.3 µM. Congeners **9**–**11** exhibited pancancer inhibitory activities across a panel of tumor cell lines, including SF-268, MCF-7, HepG-2, and A549, with IC_50_ values spanning from 13.0 to 115.3 µM [51]. In addition, compound **12** exhibited negligible cytotoxic activity, with an IC_50_ value of 49.15 µM against A549 cells [52].

#### 3.1.3. Chromones

Marine-derived *Penicillium citrinum* BCRC09F458 yielded chromone derivatives **13**–**16**, with structure–activity profiling revealing critical pharmacophoric determinants. Epiremisporine B (**16**) bearing a 2-hydroxyl moiety demonstrated dual cytotoxicity against A549 (IC_50_, 32.29 µM) and HT-29 cells (IC_50_, 50.88 µM), exhibiting enhanced potency over non-hydroxylated analogs **13**–**15**. The 2’β-methoxy-substituted epiremisporine E (**15**, IC_50_, 43.82 µM) showed greater activity against A549 cells than α‘-methoxy congeners **13** and **14** (>100 µM). Mechanistic analysis confirmed mitochondrial transmembrane potential collapse and caspase-3 activation in **15**/**16**-treated A549 cells. The SAR study established hydroxylation at C-2 and β-stereospecific methoxylation at C-2’ as critical enhancers of apoptogenic efficacy in marine chromones [52]. Epiremisporines F–H (**17**–**19**) isolated from *Penicillium citrinum* BCRC09F458 exhibited dose-dependent cytotoxicity against the HT-29 (IC_50_, 21.17–44.77 µM) and A549 (IC_50_, 31.43–77.05 µM) cell lines [53]. Structural optimization analysis revealed that epiremisporine H (**19**), bearing 4-methyl and 11-hydroxyl substitutions, demonstrated enhanced potency compared to methyl-deficient analogs **13**–**18** [52,53]. Concurrently, sponge-associated *Penicillium citrinum* SCSIO41017 yielded coniochaetone M (**20**), which showed broad-spectrum cytotoxic activity against the SF-268, MCF-7, HepG2, and A549 cell lines, with IC_50_ values ranging from 43.0 to 16.0 µM [51]. The marine-derived *Penicillium* sp. MCCC3A00228 produced *D*-arabinitol-anofinicate (**21**), a structurally unique compound demonstrating modest transcriptional activation of the orphan nuclear receptor Nur77 [54]. This receptor, encoded by NR4A1, has been identified as a critical factor in breast cancer suppression [55].

#### 3.1.4. Pyrones

Kojic acid (**22**) from *Penicillium chrysogenum* S003 exhibited negligible cytotoxicity against multiple cancer cell lines (A-549, HeLa, DU-145, HepG2, MCF-7), with all IC_50_ values > 100 µM [56]. Deep-sea *Penicillium cyclopium* SD-413 produced cyclopiumolides A and B (**23** and **24**), exhibiting moderate activity against SF126, FaDu, and TE-1 cells, with IC_50_ values ranging from 5.86 to 17.05 µM [57]. Molecular docking studies revealed a preferential binding affinity for PARP1 (PDB: 4RV6). Compound **23** formed hydrogen bonds with Gln707, Ser711, Ser714, and Asp993, while compound **24** interacted with Gly863, Ser864, Tyr896, and Ser904, which accounts for their differing bioactivities [57]. *Penicillium sumatraense* SC29 associated with *Sargassum cristaefolium* yielded penisterines C and D (**25** and **26**), with the latter showing EPC growth inhibition (IC_50_, 28.5 µg/mL) and anti-angiogenic effects in zebrafish models [58]. Deep-sea-derived *Penicillium citreonigrum* MCCC3A00169 produced pyrenocine A (**27**) and citreoviridin (**28**), both of which demonstrated significant antiproliferative activity against HeLa cells, with IC_50_ values of 5.4 µM and 0.7 µM, respectively [59]. *Penicillium chrysogenum* MCCC3A00292 afforded peniciversiol A (**29**), selectively inhibiting BIU-87 cells (IC_50_, 10.21 µM), while asperdemin (**30**) showed moderate activity against esophageal (ECA109, IC_50_ > 20 µM), bladder (BIU-87, IC_50_ > 20 µM), and hepatic (BEL7402, IC_50_ 12.75 µM) cells [60]. *Penicillium* sp. XL-01 yielded verrucosidin derivatives **31**–**35**, with **35** demonstrating broad-spectrum cytotoxicity against the HeLa, MDA-MB-231, MCF-7, MGC-803, and A-549 cell lines, with IC_50_ values ranging from 1.91 to 3.91 µM. Notably, **31** and **33** exhibited selective cytotoxicity against MGC-803 (0.96 and 1.14 µM) cells, surpassing cisplatin (1.15 –1.25 µM), while maintaining low toxicity toward NRK-52E normal cells (IC_50_, 18.3–46.7 µM) [61]. Furthermore, *Penicillum citreonigrum* XT20-134 produced 2-hydroxyl-3-pyrenocine-thio propanoic acid (**36**), showing moderate activity against the Bel 7402 and HT 1080 cell lines, with IC_50_ values of 7.63 and 10.22 µM, respectively [62].

#### 3.1.5. Fatty Acids and Esters

The marine-derived endophytic fungus *Penicillium oxalicum* 2021CDF-3, isolated from *Rhodomela confervoides*, produced oxalihexane A (**37**), which demonstrated potent anti-pancreatic carcinoma activity against PATU 8988T cells (93% inhibition at 20 µM) through Wnt5a/cyclin D1 pathway modulation, inducing apoptosis via cyclin protein dysregulation [63]. *Penicillium* sp. ZZ1750 yielded penidifarnesylin A (**38**), which exhibited glioblastoma cell line inhibition against U87MG and U251 cells, with IC_50_ values of 5.9 and 27.6 µM, respectively [64]. A deep-sea fungal co-culture of *Penicillium crustosum* PRB-2 and *Penicillium fellutanum* HDN14-323 afforded penifellutins A and B (**39** and **40**), which suppressed zebrafish larval liver cell proliferation at 10 µM. Structure–activity relationship analysis identified the dicarboxylate moiety as crucial for their anti-hepatocarcinoma potential [65]. Additionally, radiclonic acid (**41**) showed broad-spectrum cytotoxicity against the ECA109, BIU-87, and BEL-7402 carcinoma cell lines, with IC_50_ values ranging from 7.70 to 13.75 µM [60].

#### 3.1.6. Furans

*Penicillium* sp. LA032 afforded penicinone A (**42**), featuring a rare furan-fused [3,4-b].pyran-5-one scaffold, which demonstrated selective cytotoxicity against HepG2 cells (IC_50_, 3.87 µM), outperforming cisplatin in B16 cell inhibition (27.91 µM vs. 20.39 µM) [66]. DES-mutagenized *Penicillium purpurogenum* G59 produced purpurogenic acid (**43**), showing differential inhibition (52.7–78.8%) against K562, HL-60, HeLa, and BGC-823 cells at 100 µg/mL [67]. Additionally, penicillic acid (**44**) demonstrated significant cytotoxicity toward lymphoma cells (L5178Y), with an IC_50_ value of 8.9 µM [48].

#### 3.1.7. Xanthone and Benzophenones

*Penicillium chrysogenum* AD-1540 yielded chryxanthones A and B (**45** and **46**), with **45** demonstrating moderate cytotoxicity against BT-549 and HeLa cells (IC_50_, 20.4 and 23.5 µM) and **46** showing selective inhibition of the A549 cell line (20.4 µM) [68]. *Penicillium citrinum* HL-5126 produced spenibenzophenone B (**47**), which exhibited cytotoxicity against A549 cells (IC_50_, 15.7 µg/mL) [69]. Additionally, deep-sea *Penicillium chrysogenum* MCCC3A00292 afforded 3,8-dihydroxy-4-(2,3-dihydroxy-1-hydroxymethylpropyl)-1-methoxyxanthone (**48**), showing differential inhibition against ECA109 (IC_50_ > 20 μM), BIU87 (IC_50_ > 20 μM), and BEL7402 carcinoma cells (IC_50_, 15.94 µM) [60]. The novel polyketide derivative penicixanthene E (**49**) was isolated from *Penicillium* sp. GXIMD03101, demonstrating moderate anti-pancreatic carcinoma activity against SW1990 cells, with an IC_50_ value of 23.8 µM [70]. 4,4’-bond secalonic acid D (**50**), isolated from the marine-derived fungus *Penicillium pancreicum* M2013714, significantly inhibited the proliferation of 22 hepatocellular carcinoma cell lines (0.484–1.384 µM) with selective tumor targeting [71].

#### 3.1.8. Quinones

Quinones, characterized by their conjugated cyclic diketone framework featuring two carbonyl groups within an aromatic system, constitute a structurally distinctive class of marine-derived secondary metabolites with multifaceted biological roles [72]. These molecular architectures have gained prominence as privileged scaffolds in anticancer drug discovery, particularly for their adaptability in natural product-inspired targeted therapy development.

Marine-derived *Penicillium* spp. have yielded numerous bioactive naphthoquinones. *Penicillium verruculosum* XWSO1F60, isolated from a marine sponge, produced averufin (**51**), demonstrating notable HL60 cell line inhibition, with an IC_50_ value of 1.005 µM. A molecular docking study revealed strong binding affinity to α-β tubulin (1JFF, -10.2202 Kcal/mol) [73]. *Penicillium brocae* HDN12-143 afforded fumigatin chlorohydrin (**52**) and iso-fumigatin chlorohydrin (**53**), showing cytotoxicity toward HL60, with IC_50_ values of 18.63 µM and 24.83 µM, respectively [74]. Endocrocin methyl ester (**54**) and emodin (**55**) were isolated for the first time from the marine-derived fungus *Penicillium* sp. WP-13, and both exhibited negligible cytotoxic activity against the K562, BEL-7402, and SGC-7901 cell lines, with IC_50_ values ranging from 31.49 to 87.67 µM, respectively [75]. Sponge-associated *Penicillium* sp. SCSIO41015 produced frangula-emodin (**55**), exhibiting selective cytotoxic activity against human gastric cancer MGC803 cells, with an IC_50_ value of 5.19 µM [76]. Rugulosin A (**56**) demonstrated broad-spectrum cytotoxicity against QGY7701, H1299, and HCT116 cells (IC_50_, 17.6–21.2 µM) [54].

Deep-sea *Penicillium thomii* YPGA3 produced penithoketone (**57**), 3,5-dihydroxy-2-methoxy-1,4-naphthalenedione (**58**), and 2-methoxyjuglone (**59**). SAR studies revealed that **59** (IC_50_, 4.9–9.1 µM) exhibited enhanced potency over its 3-hydroxylated analog **58** across MCF7, MDAMB468, C4-2B, and C4-2B/ENZR cells [77]. Peniquinones A and B (**60** and **61**) were isolated from the marine fungus *Penicillium* sp. L129. Compound **60** showed moderate cytotoxicity against the MCF-7, U87, and PC-3 cell lines, with IC_50_ values ranging from 9.01 to 14.59 µM, while **61** exhibited relatively weaker cytotoxic activity, with IC_50_ values between 13.45 and 25.32 µM [78]. Mangrove-derived *Penicillium* sp. SCSIO41411 afforded embelin A (**62**), demonstrating cytotoxicity against PC-3 and LNCaP cells, with IC_50_ values of 18.69 and 31.62 µM, respectively [79]. Questiomycin A (**63**), isolated from the marine-derived *Penicillium* sp. ZZ1750, demonstrated moderate cytotoxicity (IC_50_, 14.13–22.56 µM) through ROS-mediated apoptosis, involving mitochondrial membrane potential reduction and caspase-3 activation [80].

#### 3.1.9. Phenolics

Phenolic compounds, characterized by aromatic rings with hydroxyl substituents, have emerged as promising Nrf2 inhibitors for cancer chemoprevention. Nrf2, a key regulator of the oxidative stress response, represents a novel therapeutic target, with natural phenolics like curcumin and resveratrol demonstrating significant anticancer potential [81,82,83,84]. This review identifies 29 phenolics with broad-spectrum anticancer activity.

##### Phenol

*Penicillium anacriticum* KMM4685 produced β-resoantarctines A and B (**64** and **66**) and 8-dehydro-β-resoantarctine A (**65**), exhibiting cytotoxic activities against human prostate cancer cell lines LNCaP, DU145, and 22 Rv1, with IC_50_ values ranging from 31 to 82.5 μM [85]. *Penicillium aculeatum* yielded novel sulfones pensulfonoxy (**67**) and pensulfonamide (**68**), and the latter demonstrated potent MCF-7 and HCT-116 cell line inhibition (IC_50_, 2.18 and 6.12 µM), while **67** showed remarkable cytotoxicity toward HCT-116 (IC_50_, 5.23 μM) [86]. *Penicillium* sp. ZZ1750 produced peniresorcinosides A–E (**69–73**), with glycosylated alkylresorcinols **69** and **70** exhibiting moderate anti-glioma activity against U87 MG and U251 cells, with IC_50_ values between 4.0 and 14.1 µM. SAR studies indicated that long-chain fatty acid substitution in **71–73** reduced potency (19.4–53.0 μM) [64]. Sorbicillin (**74**), isolated from the deep-sea fungus *Penicillium allii-sativi* MCCC3A00580, induced G2-M-phase arrest in HT-29 cells through p-H3 and cyclin B1 upregulation at concentrations between 5 and 30 μM [87]. Resorcinoside A (**75**), isolated from *Penicillium janthinellum* 168CLC-17, exhibited significant cytotoxic activity against the NUGC-3 gastric cancer cell line, with a GI_50_ value of 9.3 µM [88].

Additionally, 3,3′-dihydroxy-5,5′-dimethyldiphenyl ether (**76**) demonstrated inhibitory effects against BIU-87 cells (IC_50_, 16.41 µM), and violaceol-II (**77**) showed strong inhibitory activity against ECA 109 cells (IC_50_, 8.95 µM). Furthermore, compounds **76** and **77** exhibited moderate inhibitory potential against multiple cancer cell lines (ECA 109, BIU-87, BEL-7402), with IC_50_ values exceeding 20 µM [60]. Dicitrinone F (**78**) and phenol acid (**79**) were isolated from the marine sediment-derived fungus *Penicillium citrinum* VM6. Compound **78** displayed broad-spectrum cytotoxicity against several cancer cell lines, including A549, MCF7, MDA-MB-231, Hela, and AGS, with IC_50_ values ranging from 6.7 to 29.6 µg/mL. In contrast, **79** exhibited selective cytotoxicity against the MCF7 cell line, with an IC_50_ value of 98.1 µg/mL [89]. Spirolaxine (**80**), isolated from *Penicillium* sp. ZYX-Z-143, showed mild cytotoxicity against BEL-7402, with an IC_50_ value of 34.35 µM [90].

Xanthocillin X (**81**), isolated from the marine-derived *Penicillium* sp. ZZ1750, exhibited moderate cytotoxicity (IC_50_, 13.65–16.33 µM) through ROS-mediated apoptosis, involving mitochondrial membrane potential reduction and caspase-3 activation [80]. Marine sediment-derived *Penicillium* sp. SY2107 afforded (*Z*)-*N*-(4-hydroxystyryl)formamide (**82**), demonstrating antiproliferative activity against U251 and U87 MG cells (IC_50_, 17.0 and 39.8 µM, respectively) [91]. Halociline (**83**), obtained from *Penicillium griseofulvum*, demonstrated potent SGC-7901 and HeLa cell cytotoxicity (IC_50_, 0.870 and 1.442 µM). Network pharmacology and molecular dynamics simulations revealed high-affinity binding to MAPK1, MMP9, and PIK3CA (ΔEtotal, –20.28 to –27.94 kcal/mol), with maintained protein structural stability and enhanced interaction strength [92].

##### Phenolic Acid

*Penicillium chrysogenum* LD-201810 afforded (2’*R*)-westerdijkin A (**84**) and (*S*)-(+)-11-dehydrosydonic acid (**85**), with **84** demonstrating HepG2 cell line inhibition (IC_50_, 22.0 µM) and **85** showing differential cytotoxicity against A549 and THP-1 cells (21.2 and 18.2 µM) [93]. *Penicillium parvum* HDN17-478 produced penicacids F-G (**86–87**) alongside mycophenolic acid (**88**) and its methyl ester (**89**). Compounds **88** and **89** exhibited moderate cytotoxicity across six cancer cell lines (HCT-116/BEL-7402/MGC-803/SH-SY5Y/HO-8910/HL-60), with IC_50_ values ranging from 1.69 to 12.98 µM, while **86–87** demonstrated mild activity (IC_50_, 12.61 to 26.38 μM) [94]. *Penicillium chrysogenum* VH17 produced 2-(2-aminopropanamido)benzoic acid (**90**), showing weak MCF-7 and HepG2 cell line inhibition (87.1 and 97.3 µM) [95].

##### Phenol Ethers

Dehydrocurvularin (**91**), isolated from the mangrove-derived *Penicillium sumatrense* MA-325, exhibited moderate cytotoxicity against the HCT 116, 786-O, 5673, and Hela cell lines, with IC_50_ values ranging from 3.5 to 14.9 µM. Notably, **91** exhibited superior inhibition against bladder cancer cell line 5673 (IC_50_, 3.5 µM) compared to cisplatin (IC_50_, 4.1 µM), while maintaining low toxicity toward human embryonic kidney 293T cells (an inhibition of 54.5% at 20 µM) [96]. Additionally, 5,5-dichloro-1-(3,5-dimethoxyphenyl)-1,4- dihydroxypentan-2-one (**92**) showed moderate cytotoxicity against the Bel7402 and HT1080 cell lines (IC_50_, 13.14 and 16.53 µM, respectively) [62].

#### 3.1.10. Sorbicillinoids

Additionally, *Penicillium citrinum* SCSIO41402 afforded sorbicillfuran B (**93**), showing potent HL-60 cell line inhibition (IC_50_, 9.6 µM) [97]. Sorbicatechol D (**94**), isolated from the deep-sea fungus *Penicillium allii-sativi* MCCC3A00580, induced G2-M-phase arrest in HT-29 cells through p-H3 and cyclin B1 upregulation at 30 µM [87].

#### 3.1.11. Pyridinone Derivatives

Penipyridinone B (**95**) was isolated from the marine-derived *Penicillium* sp. ZZ1750, which exhibited moderate anti-glioma activity against U87MG and U251 cells, with IC_50_ values of 2.45 and 11.40 µM, respectively [80].

#### 3.1.12. Others

The marine-derived fungus *Penicillium citreonigrum* MCCC3A00169 produced terrein (**96**), which demonstrated potent cytotoxicity through multiple mechanisms. In HeLa cells, **96** induced apoptosis (IC_50_, 11.3 µM) and G_0_-G_1_-phase cell cycle arrest [59]. Additionally, it inhibited A549 cell proliferation while suppressing metastasis through the modulation of adhesion, migration, and invasion processes [98]. *Penicillium chrysogenum* S003 yielded the glycoside LAMA (**97**), showing limited cytotoxicity across multiple cancer cell lines (A-549/HeLa/DU-145/HepG2/MCF-7), with all IC_50_ values exceeding 100 µM [56].

### 3.2. Alkaloids

**Figure 8:**
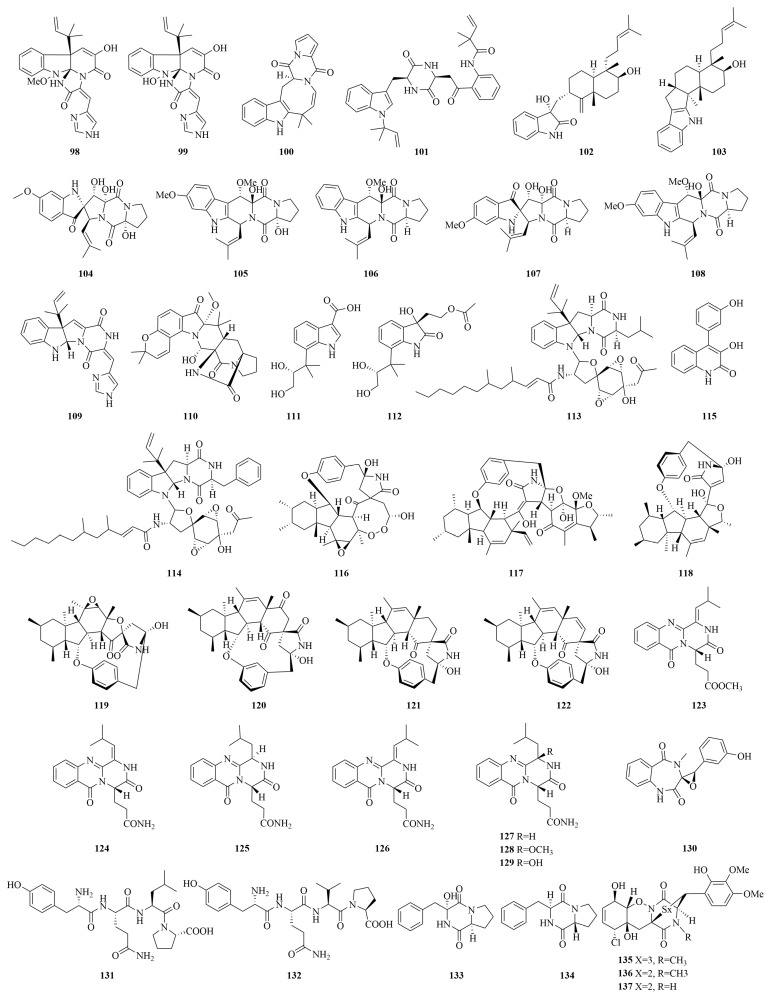
The chemical structures of cytotoxic alkaloids (**98**–**137**).

Alkaloids represent a structurally diverse class of nitrogen-containing cyclic compounds that have gained prominence in anticancer drug discovery. These natural products exert their pharmacological effects through multiple mechanisms, including topoisomerase inhibition, apoptosis induction, and the modulation of key signaling pathways involved in cell proliferation and survival [99]. The structural diversity of cytotoxic alkaloids derived from marine *Penicillium* spp. is demonstrated in Figure 8.

#### 3.2.1. Cytochalasins

Cytochalasins represent a structurally unique class of fungal metabolites featuring a tricyclic core with a macrocyclic ring fused to an isoindolinone system. These polyketide–amino acid hybrid metabolites exhibit diverse biological activities [100]. Meleagrin (**98**) and glandicoline B (**99**), isolated from the deep-sea-derived *Penicillium* sp. YPGA11, exhibited inhibitory effects against four esophageal cancer cell lines (EC109, EC9706, KYSE70, and KYSE450), with IC_50_ values ranging from 25.03 to 55.37 µM [101]. SAR analysis revealed that the methoxy group in **98** conferred enhanced potency compared to the hydroxy group in **99**, with **98** also exhibiting HepG2 cell line inhibition (IC_50_, 7.0 µM) [102].

#### 3.2.2. Indole Alkaloids

The indole scaffold, a ubiquitous heterocycle in nature, has emerged as a privileged structure in anticancer drug discovery due to its structural diversity and multifunctionality [103]. These compounds target key oncogenic pathways through histone deacetylases (HDACs), silent information regulators, PIM kinases, DNA topoisomerases, and s-receptors. Notably, 3,5-disubstituted indoles exhibit promising PIM kinase selectivity, with C-3 and C-5 modifications enabling isoform-specific affinity modulation [103,104]. This review highlights 15 indole derivatives from marine *Penicillium* spp., offering insights for targeted molecular modifications in anticancer drug development.

*Penicillium dimorphosporum* KMM4689 yielded deoxy-14,15-dehydroisoaustamide (**100**), the first deoxyisoaustamide alkaloid featuring a doubly unsaturated proline ring. At non-cytotoxic concentrations, **100** specifically degrades AR-V7, resensitizing resistant prostate cancer cells to enzalutamide, thus enhancing AR-targeted therapy efficacy [105]. The deep-sea-derived fungus *Penicillium* sp. LSH-3-1 yielded a new compound, peniokaramine (**101**), which displayed cytotoxic activity against the A549 cell line, showing an inhibition rate of 53.43% at 50 µM [106]. Penicindopene A (**102**), a novel indole diterpene, isolated from *Penicillium* sp. YPCMAC1, exhibited moderate cytotoxicity against A549 and HeLa cells, with IC_50_ values of 15.2 µM and 20.5 µM, respectively [107]. Additionally, emindole SB (**103**), obtained from *Penicillium* sp. KFD28, demonstrated moderate cytotoxic effects against K562 cells, with an IC_50_ value of 18.8 µM [108].

From the marine fungus *Penicillium brasilianum* HBU-136, spirotryprostatin G (**104**), cyclotryprostatins F and G (**105** and **106**), spirocyclic diketopiperazine alkaloid (**107**), and cyclotryprostatin B (**108**) were isolated. Compounds **104** and **107** showed selective HL-60 cell inhibition (IC_50_, 6.0 and 7.9 µM), while **105**, **106**, and **108** demonstrated MCF-7 cytotoxicity (IC_50_, 5.1–10.8 µM) [109]. Deep-sea *Penicillium granulatum* MCCC3A00475 yielded roquefortine J (**109**), exhibiting HepG2 cell line inhibition (IC_50_, 19.5 µM) [102]. The OSMAC-based cultivation of *Penicillium oxalicum* 2021CDF-3, an endophyte of marine red algae, yielded asperinamide B (**110**) and peniochroloids A–B (**111**–**112**). Compound **110**, featuring a rare 3-pyrrolidone moiety, demonstrated potent human pharyngeal squamous FADU cell line inhibition (IC_50_, 0.43 µM), comparable to doxorubicin (IC_50_, 0.07 µM). SAR analysis revealed that the ester carbonyl group in **112** enhanced A549 cytotoxicity (IC_50_, 15.30 µM) compared to **111** (IC_50_, 29.84 µM) [110]. Neomycin-resistant *Penicillium purpurogenum* G59 mutant strain 3-f-31 yielded penicimutanins C (**113**) and A (**114**), exhibiting broad-spectrum cytotoxicity against the K562, HL-60, HeLa, BGC-823, and MCF-7 cell lines with IC_50_ values between 5.0 and 11.9 µM [111].

#### 3.2.3. Pyridine Alkaloids

Viridicatol (**115**), isolated from the deep-sea fungus *Penicillium solitum* MCCC3A00215, exhibited moderate cytotoxic activity against PANC-1, HeLa, and A549 cells, with IC_50_ values of 18, 19, and 24 µM, respectively [112].

#### 3.2.4. Pyrrole and Pyrrolidine Alkaloids

*Penicillium citrinum* DY180712 produced perpyrrospirone A (**116**), featuring an unprecedented 6/5/6/8/5/13/6 oxahexacyclic scaffold with a peroxide-bridged 8,9-dioxa-2-azaspiro [4.7]dodecane core, alongside penicillione G (**117**). Both compounds demonstrated broad-spectrum cytotoxicity against six human tumor cell lines (MGC803/HepG2/MDA-MB-231/MCF-7/Bel-7402/HeLa), with IC_50_ values ranging from 2.5 to 38.9 µM [113]. Crab-associated *Penicillium* sp. ZZ380 yielded penicipyrroether A (**118**) and pyrrospirone J (**119**), featuring a unique 6/5/6/5 fused ring system. These compounds exhibited potent glioblastoma U87MG and U251 cell line inhibition, with IC_50_ values ranging from 1.64 to 17.92 µM [114]. Additionally, pyrrospirone G (**120**) demonstrated significant anti-glioma activity across multiple cells (U87MG/U251/SHG44/C6, IC_50_, 1.06–8.52 µM), while pyrrospirones H and I (**121**–**122**) showed moderate activity (IC_50_, 7.44–26.64 µM) [115].

#### 3.2.5. Quinazolines and Their Analogs

*Penicillium polonicum* MN623481 produced polonimides A–C (**123**–**125**) alongside known quinazoline-containing piperazine diketones aurantiomides A–C (**126**, **128**, **129**) and anacine (**127**). Compound **127** demonstrated notable cytotoxicity against the A549, HGC-27, and UMUC-3 cell lines (IC_50_, 6.0–7.2 µM) [116]. Cyclopenol (**130**) exhibited selective cytotoxicity against BIU-87 and BEL-7402 cells (IC_50_, 8.34 and 7.81 µM, respectively) [60].

#### 3.2.6. Peptides

*Penicillium citrinum* 2015PF07, cultured with ScCl_3_ (50 µM), produced novel compounds **131** and **132**, demonstrating selective cytotoxicity against the HCT-115 and MCF-7 cell lines (IC_50_, 20 µg/mL) [117]. Tunicate-associated *Penicillium* sp. from the Red Sea yielded penicillatides B (**133**) and cyclo (*R*-Pro-*S*-Phe) (**134**), exhibiting selective HCT-116 cell line inhibition (IC_50_, 6.0 and 9.57 μg/mL, respectively) [118].

#### 3.2.7. Thiodiketopiperazines

*Penicillium ludwiglium* SCSIO41408 afforded adametizines C (**135**) and A (**136**), along with DC1149B (**137**). These compounds exhibited 22Rv1 prostate cancer cell line inhibition (IC_50_, 13.0–13.9 µM), with **137** showing notable PC-3 cell cytotoxicity (IC_50_, 5.1 µM) and dose-dependent apoptosis induction [119].

### 3.3. Terpenoids

**Figure 9:**
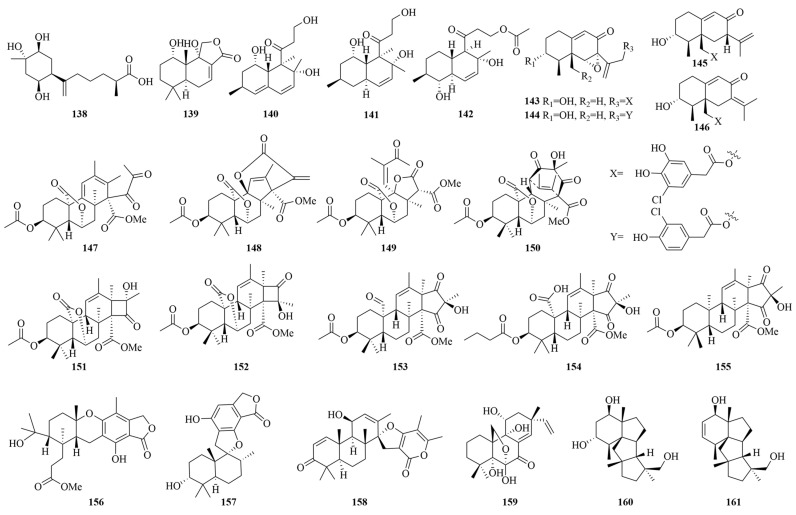
The chemical structures of anticancer terpenoids (**138–161**).

Terpenoids, the largest and most structurally diverse class of natural products, are biosynthesized via the mevalonic acid pathway with isoprene units serving as fundamental building blocks. Notably, marine-derived sesquiterpenoids exhibit diverse biological activities and contribute to approximately one-third of reported cytotoxic compounds within this chemical class [120]. Their pharmacological potential is underscored by multifaceted anticancer mechanisms, including antiproliferative, apoptotic, anti-angiogenic, and antimetastatic activities, making them particularly intriguing for drug discovery [121,122]. The structural diversity of anticancer terpenoids derived from marine *Penicillium* spp. is displayed in Figure 9.

#### 3.3.1. Sesquiterpenes

*Penicillium puericum* MZY-202312-521 yielded inonotic acid C (**138**), demonstrating significant MCF-7 breast cancer cell line inhibition (IC_50_, 7.7 µM) [123]. Purpuride G (**139**), isolated from the marine fungus *Penicillium minioluteum* ZZ 1657, demonstrated moderate inhibitory effects on glioblastoma U251 and U87 MG cells, with IC_50_ values of 4.49 and 10.9 µM, respectively [124]. Additionally, decumbenones A and B (**140** and **141**) demonstrated ECA109 cell line inhibition (IC_50_, 12.41 and 15.60 µM, respectively) [60]. Seagrass-associated *Penicillium yezoense* KMM4679 produced 1-acetylpallidopenilline A (**142**), exhibiting potent MCF-7 cell line inhibition (IC_50_, 0.66 µM). Structure–activity relationship analysis revealed that the 1-acetyl chain is crucial for enhancing cytotoxic activity [125]. *Penicillium copticola* WZXY-m122-9 produced copteremophilanes D(**143**), E(**144**), G(**145**), and H (**146**), with **146** exhibiting selective A549 cell inhibition (IC_50_, 3.23 µM). SAR analysis revealed that C-7/C-11 olefin rearrangement in **145** reduced potency (IC_50_ > 10 µM), while C-12 phenylacetic acid substitution in **143** and **144** enhanced HCT-8 cell line inhibition (IC_50_, 5.4–7.3 µM) [126].

#### 3.3.2. Meroterpenoids

Meroanapartines A–C (**147**–**149**), featuring unprecedented 6/5/6/6, 6/5/6/5/6, and 6/5/6/5 polycyclic systems, were isolated from the marine fungus *Penicillium anacuticum* KMM 4685. These compounds exhibit *P*-glycoprotein (*P*-gp)-inhibitory activity, resensitizing drug-resistant cancer cells to docetaxel and serving as promising leads for combination therapies [127]. *Penicillium* sp. SCSIO41512 produced penicimeroterpenoids A–C (**150–152**), featuring unprecedented 5/6/6/6/7 and 5/6/6/6/4 fused ring systems. These compounds demonstrated CDC25B phosphatase inhibition (IC_50_, 20 µM) [128]. *Penicillium* sp. A18 yielded penimeroterpenoid A (**153**), showing weak cytotoxicity against A549, HCT116, and SW480 cells (IC_50_, 78.63–95.54 µM) [129]. Penisimplinoid F (**154**), isolated from *Penicillium simplicissimum* 19XS15ZM-3, displayed notable cytotoxic activity against NCI-H446 cells (IC_50_, 6.49 µM) [130]. Andrastone A (**155**), obtained from the deep-sea fungus *Penicillium allii-sativi* MCCC3A00580, selectively inhibited HepG2 cells (IC_50_, 7.8 µM) through caspase-3 activation and RXRα modulation [131]. The deep-sea-derived *Penicillium thomii* YPGA3 produced the new austalide meroterpenoid **156**, which displayed mild breast cancer cytotoxicity against MDA-MB-468 cells, with an IC_50_ value of 38.9 µM [132]. Stachybotrylactone B (**157**), isolated from the soft coral-associated *Penicillium* sp. SCSIO41201, demonstrated broad-spectrum cytotoxicity against leukemia (HL-60/K562/MOLT-4) and renal carcinoma (ACHN/786-O/OS-RC-2) cell lines, with IC_50_ values ranging from 4.12 to 23.55 µM [133]. Marine-derived *Penicillium* sp. TJ403-1 yielded brevione I (**158**), exhibiting notable cytotoxicity against the HL-60, A-549, and HEP3B cell lines (IC_50_, 4.92–8.60 µM). The α,β-unsaturated carbonyl moiety in its structure, hypothesized to form covalent bonds with cysteine thiol groups, is crucial for its bioactivity [134,135]. 

#### 3.3.3. Diterpenoids

*Penicillium sclerotiorum* GZU-XW03-2 afforded diaporthein B (**159**), which significantly inhibited HCT116 and LOVO colorectal cancer cell proliferation and migration (IC_50_, 1.5 and 3.0 µM, respectively) while promoting apoptosis. Mechanistic studies revealed that **159** modulates the Hippo/YAP/TAZ and TP53/BCL-2/BAX pathways, impairing mitochondrial function in cancer cells without significantly affecting normal intestinal epithelial cells [136]. Additionally, deep-sea-derived *Penicillium* sp. YPGA11 afforded conidiogenol D (**160**) and conidiogenone C (**161**), which demonstrated inhibitory effects on five esophageal cancer cell lines (EC109, EC9706, KYSE30, KYSE70, and KYSE450), with IC_50_ values ranging from 27.05 to 54.7µM [101].

### 3.4. Steroids

The steroid 22-triene-3,5-diol (**162**), discovered from *Penicillium leveling* N33.2, showed cytotoxic activity against HepG2, A549, and MCF-7 cancer cells (IC_50_, 2.89–18.51 µg/mL) alongside pancreatic lipase and α-glucosidase inhibition [137]. Deep-sea *Penicillium solarum* MCCC3A00215 yielded solitumergosterol A (**163**), a C30 steroid with a 6/6/6/6/5 pentacyclic skeleton showing MDA-MB-231 cell inhibition (44.1% at 20 µM) [138]. Ergosterol (**164**) and epidioxyergosterol (**165**), isolated from *Penicillium chrysogenum* strain S003, exhibited moderate cytotoxic activity against the A549, DU-14, MCF-7, and HepG 2 cell lines, with IC_50_ values ranging from 2.89 to 21.26 µM [56].

**Figure 10:**
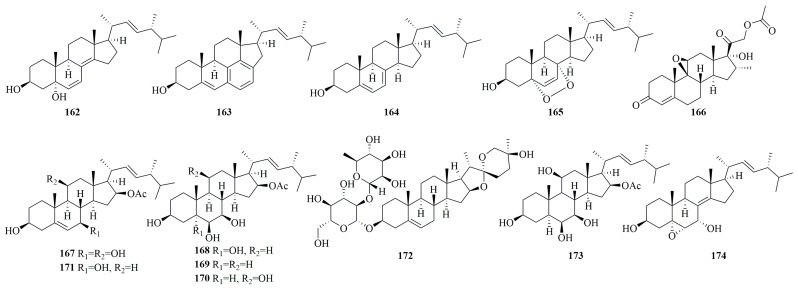
The chemical structures of anticancer steroids (**162–174**).

16α-Methylpregna-17α,19-dihydroxy-(9,11)-epoxy-4-ene-3,18-dione-20-acetoxy (**166**), derived from the sponge-associated *Penicillium citrinum* SCSIO41017, exhibited moderate cytotoxic activity against four tumor cell lines (SF-268, MCF-7, HepG-2, and A549), with IC_50_ values ranging from 13.5 to 18.0 µM [51]. Penicisteroids E (**167**), G (**168**), H (**169**), A (**170**), and C (**171**), isolated from the deep-sea fungus *Penicillium granulatum* MCCC3A00475, demonstrated selective cytotoxicity against A549, BIU-87, BEL-7402, ECA-109, Hela-S3, and PANC-1 cells, with IC_50_ values between 4.1 and 14.4 µM. Further studies revealed that compounds **167** and **170** could induce apoptosis via an RXRα-dependent mechanism by modulating the transcriptional expression of retinoid X receptor (RXR) α and promoting the cleavage of poly (ADP-ribose) polymerase (PARP), acting as effective RXRα binders with Kd values of 13.8 and 12.9 µM, respectively [139]. Additionally, isonuatigenin I (**172**) and penicisteroid A (**173**) demonstrated HepG2 cell line inhibition (IC_50_, 8.6 and 8.2 µM, respectively) [102], while 5α,6α-epoxy-(22*E*,24*R*)-ergosta-8(14),22-diene-3β,7α-diol (**174**) showed mild cytotoxicity against HepG2, A549, and MCF7 cells (IC_50_, 29.4–36.72 µM) [95]. The molecular architectures of anticancer peptides derived from marine *Penicillium* spp. are delineated in Figure 10.

The cytotoxic compounds isolated from marine *Penicillium* spp., including their names, numbers, source, biological activities, and associated references, are comprehensively summarized in Table 1.

## 4. Drugability Assessment

### 4.1. Dicitrinones G

The tangeretin dimer dicitrinone G (**8**) has exhibited potent antiproliferative effects against pancreatic cancer through multimodal mechanisms, addressing the critical clinical challenge of limited therapeutic options for this malignancy. In cytotoxicity evaluations using doxorubicin hydrochloride as the positive control (IC_50_, 18.24 μM), dimer **8** demonstrated superior activity toward BxPC-3 cells (IC_50_, 12.25 μM), indicating enhanced pharmacological efficacy compared to conventional chemotherapeutics [50,140]. Notably, its cytotoxic profile in PANC-1 cells (IC_50_, 24.33 μM) paralleled that of doxorubicin (IC_50_, 24.00 μM), suggesting cell line-dependent therapeutic variations [50,140]. In xenograft tumor models, **8** combined with 5-fluorouracil (5-FU) showed no toxicity in major organs (heart, liver, spleen, lungs, kidneys), indicating superior safety compared to tangeretin [141]. Mechanistic studies revealed that **8** inhibits pancreatic cancer angiogenesis via the Notch1 signaling pathway, as evidenced by reduced CD31 expression in tumor tissues. Additionally, in human umbilical vein endothelial cells (HUVECs), **8** suppressed proliferation and angiogenesis, further supporting its anti-tumor-vascularization effects [140] Under hypoxic conditions, **8** reduced interleukin-18 (IL-18) levels in a BXPC-3 cell culture medium. The combination of IL-18 blocking agent (IL-18 BP) with the hypoxic BXPC-3 conditioned medium (CM) confirmed that **8** inhibits pancreatic cancer angiogenesis by suppressing IL-18 release. Furthermore, **8** decreased NLRP3 inflammasome activation, as shown by reduced NLRP3 and caspase-1 p20 expression in tumor tissues treated with **8** and 5-FU. Genetic knockout of NLRP3 in BXPC-3 cells (gNLRP3) demonstrated that **8** inhibits angiogenesis by suppressing NLRP3 inflammasome assembly [140]. Given that NLRP3 inflammasome activation and IL-18 production are critical in pancreatic cancer progression [142,143] and considering IL-18’s role as a CXCR1/2 ligand promoting tumorigenesis and angiogenesis [144,145,146,147], **8** represents a promising marine-derived therapeutic candidate. Its ability to inhibit IL-18/NLRP3-regulated NICD suggests potential for microvascular-targeted pancreatic cancer treatment strategies [140].

### 4.2. Penitrem A

**Figure 11:**
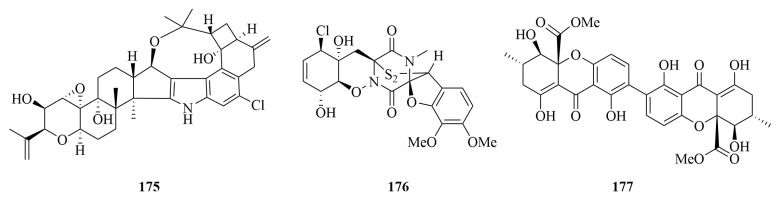
The chemical structures of compounds **175–177**.

Penitrem A (**175**), an indole diterpenoid alkaloid (Figure 11) from marine-derived *Penicillium commune* GS20, demonstrates potent antiproliferative, antimigratory, and anti-invasive bioactivities against human breast cancer cells. This compound induces G1-phase cell cycle arrest by upregulating the cyclin-dependent kinase inhibitor p27 [148,149]. As a novel Wnt/β-catenin pathway inhibitor, **175** significantly reduces total β-catenin expression in MDA-MB-231 cells, effectively blocking breast cancer cell proliferation and migration both *in vitro* and *in vivo* [150,151,152]. Recent advances in Wnt/β-catenin-targeted drug development have highlighted the clinical translatability of such inhibitors. Notably, E7386—an orally bioavailable and selective inhibitor disrupting the β-catenin/CREB-binding protein (CBP) interaction—has emerged as a promising agent. Preclinical studies revealed that E7386 blocks Wnt/β-catenin signaling in HEK 293 cells and APC-mutant human gastric cancer ECC10 cells, with minimal off-target effects [153]. These findings align with the growing recognition of β-catenin/CBP complex inhibitors as a viable strategy to circumvent limitations of upstream Wnt pathway targeting, particularly in tumors driven by APC mutations.

Additionally, **175** functions as a BK channel antagonist, exhibiting antiproliferative effects in BK channel-overexpressing breast cancer subtypes. It synergistically enhances the efficacy of HER-targeted drugs (lapatinib and gefitinib) through STAT3 and p27 pathway modulation, offering promising strategies to overcome targeted therapy resistance [149]. These multifaceted mechanisms position **175** as a promising lead compound for breast cancer therapeutics, particularly in overcoming drug resistance and enhancing targeted therapy sensitivity.

### 4.3. Penicisulfuranol A

Penicisulfuranol A (**176**), an epipolithiodioxopiperazine (ETP) alkaloid (Figure 11) from mangrove-derived *Penicillium janthinellum* HDN13-309, demonstrated potent cytotoxicity against HeLa and HL-60 cells, with IC_50_ values of 0.5 µM and 0.1 µM, respectively. Notably, its potency was comparable to the positive control adriamycin (IC_50_ = 0.5 µM and 0.2 µM for HeLa and HL-60, respectively) [154]. Mechanistic studies revealed that **176** is a novel C-terminal inhibitor of heat shock protein 90 (Hsp90), disrupting its molecular chaperone function independent of the ATP-binding domain. As a key regulator of over 200 client proteins involved in cell growth signaling, Hsp90 represents a promising anticancer target. The clinical validation of this approach was recently demonstrated by the 2022 approval of pimitespib (Jeselhy^®^) by Taiho Pharmaceutical, an oral Hsp90α/β inhibitor for chemotherapy-refractory gastrointestinal stromal tumors (GISTs) [155]. Unlike pimitespib and other N-terminal inhibitors, **176** significantly reduced Hsp90 client protein levels without inducing Hsp70 expression, inducing apoptosis in HCT116 cells *in vitro* and *in vivo* while inhibiting xenograft tumor growth. Compound **176** inhibits Hsp90 C-terminal dimerization, prevents ADH protein disaggregation, and disrupts co-chaperone interactions. The disulfide bond in **176** was identified as crucial for Hsp90 inhibition, binding to cysteine residues near the amino acid region [156]. Extensive synthetic studies have focused on spirocyclic diketopiperazine intermediates of **176** [157]. As a novel Hsp90 C-terminal inhibitor, **176** represents a promising lead for investigating Hsp90 biology and developing colorectal cancer therapeutics.

### 4.4. Secalonic Acid D

Secalonic acid D (SAD, **177**), a polyketide mycotoxin produced by the marine-derived fungus *Penicillium oxalicum*, exhibited representative bioactivity among secondary metabolites from marine fungi. Pharmacological studies demonstrated its potent cytotoxicity against K562, A549, and P388 tumor cell lines (IC_50_, 0.03–5.76 µM), while showing significantly weaker activity against BEL-7402 hepatoma cells (IC_50_, 15.50 µM), indicating remarkable tumor-selective efficacy. Notably, its effective dose in murine models was over 50-fold lower than the teratogenic threshold, highlighting a favorable therapeutic window [158,159]. Further investigations revealed potent cytotoxicity of **177** against HL-60 and K562 cells, with IC_50_ values of 0.38 µM and 0.43 µM, respectively. Mechanistically, **177** enhanced the kinase activity of glycogen synthase kinase-3β (GSK-3β) by promoting dephosphorylation at Ser9, thereby triggering ubiquitin-proteasome-dependent degradation of β-catenin. This process disrupted the transcriptional activation of the Wnt/β-catenin signaling pathway, downregulated the oncogene c-Myc, and ultimately induced G1-phase cell cycle arrest in HL-60 and K562 leukemia cells [160]. Additionally, **177** acted as a novel DNA topoisomerase I (topo I) inhibitor via a non-covalent binding mechanism, with a minimum inhibitory concentration (MIC) of 0.4 µM. Unlike camptothecin (CPT), which stabilized topo I-DNA covalent complexes to induce DNA damage, **177** selectively blocked the dynamic interaction between topo I and DNA substrates, thereby mitigating genomic toxicity risks. Its dose-dependent inhibition correlated with molecular conformational flexibility, target-binding affinity, and intracellular metabolic stability [161]. The non-covalent mode of action positions **177** as a promising candidate for optimized drug delivery systems, such as nanocarriers or prodrug designs, to enhance tumor-targeted efficacy while reducing off-target effects.

### 4.5. 4,4′-Bond Secalonic Acid D

4,4’-Bond secalonic acid D (**50**), a marine fungal metabolite, demonstrated notable antiproliferative activity across 22 human cancer cell lines (BGC-823, SGC-7901, HGC-27, EC9706, KYSE450, CNE1, CNE2, SW620, SW480, LOVO, HuH-7, PLC/PRF/5, SK-HEP, HeLa, A549, SK-MES-1, SPC-A1, 95D, Jeko-1, Raji, U937, A375, HFF, H22) with IC_50_ values ranging from 0.484 to 1.384 µM [71]. Notably, **50** showed preferential cancer cell targeting with minimal cytotoxicity against normal human foreskin fibroblasts (HFFs). Mechanistic studies revealed that **50** induced mitochondria-mediated apoptosis by modulating the Bcl-2/Bax protein ratio. It also inhibited side population (SP) cell growth in hepatocellular carcinoma models (PLC/PRF/5, HuH-7) by downregulating ATP-binding cassette superfamily G member 2 (ABCG2) expression. Furthermore, **50** suppressed SP cell invasion and migration through matrix metalloproteinase-9 (MMP-9) downregulation and metalloproteinase-1 (TIMP-1) upregulation. *In vivo* experiments using the H22 hepatocellular carcinoma mouse model confirmed its anti-lung metastasis effects by suppressing MMP-9 suppression [162]. Its mechanistic versatility contrasts with yet complements targeted agents like venetoclax—a clinically validated BCL-2 inhibitor that achieved therapeutic efficacy in acute myeloid leukemia (AML) through selective apoptotic induction. While venetoclax has demonstrated success in both preclinical xenograft models and clinical settings, its application remains restricted to hematological malignancies [163]. In contrast, **50** exhibited a broader anticancer spectrum, effectively targeting solid tumors like HCC while maintaining favorable selectivity between malignant and normal cells—a critical advantage given the dose-limiting toxicities observed with venetoclax in non-leukemic tissues.

## 5. Discussion

### 5.1. Chemical Diversity and Structural Features of Marine-Derived Penicillium Metabolites

Polyketides (**1**–**97**, **177**) constitute the largest class (55.4%) of secondary metabolites from the *Penicillium* genus, exhibiting anticancer, antibacterial, antiviral, and antioxidant properties. Phenolics, representing 29.6% of polyketides, demonstrate moderate-to-potent cytotoxicity against the HL-60, HCT-116, HCT-8, and A549 cell lines. These compounds inhibit Nrf2 nuclear translocation, reducing tumor cell resistance to chemotherapy. Their combination with other anticancer agents enhances sensitivity through Nrf2 pathway inhibition and HO-1 downregulation, highlighting their potential as chemopreventive agents or combination therapies [82,164]. Alkaloids (**98**–**137, 175, 176**), characterized by nitrogen, sulfur, and oxygen heteroatoms, often exhibit anticancer activity through DNA interactions. Indole alkaloids dominate this class, with 50% being di- or trisubstituted, primarily at C2 and C3 positions. Pyrrolidine-based trisubstituted alkaloids show significant cytotoxicity against glioma and gastric cancer cells. Metal complexes of alkaloids have emerged as potent anticancer agents, with nitrogen atoms enabling multiparameter optimization and pharmacological enhancements exceeding 1000-fold in some cases [165,166,167]. Triterpenoids (**138**–**161**), typically featuring α-methylene-γ-lactone structures, exhibit mild anticancer activity by modulating redox balance and signaling pathways, leading to cell cycle arrest and apoptosis induction [168]. Steroids (**162**–**174**) exhibit selective cytotoxicity against malignant cells, showing potential applications in hormone-based therapies, cancer vaccine development, and synergistic combination regimens. Furthermore, steroid–drug conjugates significantly enhance therapeutic efficacy in anticancer treatment through three synergistic mechanisms: improved target specificity, enhanced cellular delivery capabilities, and optimized therapeutic safety profiles, while concurrently exerting multi-pathway therapeutic effects through the strategic modulation of key cellular signaling pathways—consequently reducing systemic toxicity relative to their non-conjugated counterparts [169]. Despite their potential, many marine-derived compounds face challenges in clinical translation due to toxicity or poor bioavailability. Strategies such as nanoparticle delivery, antibody–drug conjugates, and network-based multi-target drug design are essential for enhancing stability, specificity, and efficacy [170,171,172,173].

### 5.2. Current Status of Marine Drug Development

Marine-derived antitumor drugs have gained significant attention, with several compounds advancing to clinical trials. BG136, a β-1,3/1,6-glucan from *Durvillaea antarctica*, activates innate immunity through carbohydrate receptor binding, demonstrating broad-spectrum antitumor effects [174,175]. Trabectedin (Yondelis^®^), derived from *Ecteinascidia turbinata*, is approved for soft tissue sarcoma and liposarcoma, with recent studies showing improved progression-free survival in combination with doxorubicin [176,177,178]. To date, multiple marine-derived drugs have been approved for cancer, cardiovascular diseases, and diabetes, underscoring the field’s achievements. However, the vast potential of marine natural products remains underexplored, with numerous compounds awaiting further investigation [179].

### 5.3. Challenges and Future Directions

Marine drug discovery faces significant hurdles, including the sparse distribution of medicinal organisms, low concentrations of active components, and complex extraction processes. The lack of efficient high-throughput screening methods further limits the discovery of novel compounds. Additionally, the intricate mechanisms and toxicity profiles of marine-derived substances necessitate extensive pharmacological and toxicological studies, making the development process time-consuming and costly [180].

Future research should focus on artificial cultivation, marine biobanks, and biosynthetic pathway exploration to enable sustainable resource utilization. High-throughput screening, combined with biosynthetic and chemical synthetic methods, will enhance the efficiency of bioactive compound discovery. Advanced technologies such as artificial intelligence and machine learning can optimize compound structures and predict bioactivities, accelerating drug development and deepening mechanistic understanding [181]. Interdisciplinary integration and innovative 3D cell culture models will improve screening mechanisms, while automation and software advancements will promote the development of marine-derived therapeutics, ensuring that their full potential is realized [182].

The exploration of biosynthetic gene clusters (BGCs) as reservoirs of novel bioactive molecules has established genome mining as a cornerstone of marine natural product discovery [183]. Advances in high-throughput sequencing have catalyzed exponential growth in marine genomic datasets, enabling the precise identification of signature biosynthetic genes and enzymatic domains through multi-sequence alignment and conserved structural motif analysis [184,185]. Notably, over 70% of marine-derived BGCs remain transcriptionally silent under standard laboratory conditions, necessitating activation through environmental or metabolic cues. To address this challenge, multidimensional activation strategies have emerged: (1) microenvironmental stress induction via physicochemical parameter optimization (e.g., salinity gradients, temperature shifts); (2) microbial co-culturing to simulate ecological interactions; and (3) epigenetic modulation using histone deacetylase inhibitors or chromatin remodelers. These approaches, integrated with comparative genomics, have successfully unlocked cryptic clusters, yielding marine polyketides, nonribosomal peptides, and hybrid compounds with unprecedented scaffolds, exemplified by the discovery of 95 novel compounds from marine actinomycetes and fungi in recent studies [186,187].

Bioinformatic tools, particularly hidden Markov model (HMM)-based algorithms, now achieve >90% accuracy in BGC prediction [188]. Coupled with metabolic network reconstruction, these tools elucidate module organization and substrate specificity, as demonstrated by the identification of 4000+ novel BGCs in the Ocean Microbiomics Database (OMD), including ribosomally synthesized and post-translationally modified peptides (RiPPs) from uncultivated marine Candidatus Eremiobacterota [189]. Synthetic biology further amplifies this potential: CRISPR-Cas9-mediated promoter engineering and large DNA fragment assembly enable heterologous BGC reconstitution [190]. For instance, the EQCi dynamic regulation system in Streptomyces enhanced rapamycin production by 660% [191], while the modular refactoring of fungal terpene clusters in Aspergillus oryzae accelerated the discovery of anti-inflammatory mangicol J [192]. Such integration of “BGC–metabolic flux” not only deciphers evolutionary drivers of marine microbial secondary metabolism but also establishes a foundation for combinatorial biosynthesis [185,193].

Future directions emphasize spatiotemporal pathway activation via synthetic regulatory libraries and biosensor-coupled dynamic systems. To mitigate cytotoxicity, metabolic engineering optimizes precursor supply, as evidenced by riboflavin cofactor engineering boosting caerulomycin A production 14.6-fold [194]. For bioavailability challenges, nanocarrier delivery systems—such as DNA-barcoded renal-targeting nanoparticles demonstrated in recent studies—offer solutions to overcome efflux transporters like P-gp, as seen in oral paclitaxel formulations [127]. These interdisciplinary strategies bridge discovery and scalable biomanufacturing, positioning marine natural products as sustainable drug development pipelines.

## 6. Conclusions and Future Perspectives

This review highlights 177 marine-derived anticancer secondary metabolites from *Penicillium* spp. identified between 2018 and 2024. Predominantly isolated from marine sediments, corals, sponges, and sea water, these compounds were primarily sourced from *Penicillium citrinum*, *Penicillium chrysogenum*, and *Penicillium granulatum*. This review systematically examines their chemical diversity, anticancer activities, and pharmacological potential, with polyketides and alkaloids constituting over 50% of the identified structures, followed by terpenoids and steroids. All compounds demonstrated cytotoxic activity, with dicitrinone G (**8**), 4,4’-bond secalonic acid D (**50**), penitrem A (**175**), penicisulfuranol A (**176**), and secalonic acid D (**177**) exhibiting particularly promising drug-like properties and unique mechanisms of action. While detailed anticancer data remain limited for many compounds, their structural diversity underscores their potential as scaffolds for novel drug design or combination therapies. This compilation provides a valuable resource for selecting candidates for further investigation and emphasizes the untapped potential of marine-derived natural products in anticancer drug discovery. Future research should leverage advanced technologies such as high-throughput screening, synthetic biology, and artificial intelligence to accelerate the exploration of these marine resources and elucidate their pharmacological mechanisms.

## Figures and Tables

**Table 1:** List of compounds with anticancer activity in marine *Penicillium* spp.

Compounds	Source	Producing Strain	Cell Lines	IC_50_/GI_50_/Inhibition Rate/Mechanism	References
Penidioxolane C (**1**)	Marine sponge *Holoxea* sp.	*Penicillium sclerotiorum* E23 Y-1A	K562, BEL-7402, SGC-7901, A549, Hela	23.94–60.66 µM	[46]
8a-epi-hypocrellone A (**2**)	Marine alga *Grateloupia* sp.	*Penicillium sclerotiorum*	SH-SY5Y	35.6 µM	[47]
8 α-epi-eupenicilazaphilone C (**3**)				73.2 µM	
Hypocrellone A (**4**)				>100 µM	
Eupenicilazaphilone C (**5**)				95.2 µM	
Brominated azaphilone A (**6**)	Marine sponge *Agelas oroides*	*Penicillium canescens*	/	/	[48]
Brominated azaphilone B (**7**)			L5178Y, A2780	8.9, 2.7 µM	
Dicitrinones G (**8**)	Starfish	*Penicillium* sp. GGF 16 -1-2	BXPC-3, PANC-1	12.25, 24.33 µM	[50]
Xerucitrinin A (**9**)	Sponge *Callyspongia* sp.	*Penicillium citrinum* SCSIO 41017	SF-268, MCF-7, HepG-2, A549	13.0–115.3 µM	[51]
Xerucitrinic acid A (**10**)					
Penicitrinol B (**11**)					
Penicitrinone A (**12**)			MCF-7	1.3 µM	
Epiremisporine A (**12**)	Wastewater	*Penicillium citrinum* BCRC 09F458	A549	49.15 µM	[52]
Epiremisporine C (**13**)	Wastewater	*Penicillium citrinum* BCRC 09F458	A549	>100 µM	[52]
Epiremisporine D (**14**)					
Epiremisporine E (**15**)			A549	43.82 µM	
Epiremisporine B (**16**)			A549, HT-29	32.29, 50.88 µM	
Epiremisporine F (**17**)	Wastewater	*Penicillium citrinum* BCRC 09F458	HT-29, A549	44.77, 77.05 µM	[53]
Epiremisporine G (**18**)				35.05, 52.30 µM	
Epiremisporine H (**19**)				21.17, 31.43 µM	
Coniochaetone M (**20**)	Sponge *Callypongia* sp.	*Penicillium citrinum* SCSIO 41017	SF-268, MCF-7, HepG-2, A549	43.0–16.0 µM	[51]
D-arabinitol-anofinicate (**21**)	Sea water	*Penicillium* sp.	/	Transcriptional activation of orphan nuclear receptor Nur77	[54]
Kojicacid (**22**)	Deep-sea sediment	*Penicillium chrysogenum* strain S003	A-549, HeLa, DU-145, HepG2, MCF-7	>100 µM	[56]
Cyclopiumolide A (**23**)	Deep-sea sediment	*Penicillium cyclopium* SD-413	SF126, FaDu, TE-1	5.86–17.05 µM	[57]
Cyclopiumolide B (**24**)					
Penisterine C (**25**)	Marine brown alga *Sargassum cristaefolium*	*Penicillium sumatraense* SC 29	EPC	28.5 µg/mL	[58]
Penisterine D (**26**)			/	Anti-angiogenic effects	
Pyrenocine A (**27**)	Deep-sea sediment	*Penicillium citreonigrum*	HeLa	5.4 µM	[59]
Citreoviridin (**28**)				0.7 µM	
Peniciversiol A (**29**)	Deep-sea sediment	*Penicillium chrysogenum*	BIU-87	10.21 µM	[60]
Asperdemin (**30**)			ECA 109, BIU-87, BEL-7402	>20, 12.75 µM	
Nordeoxyverrucosidin (**31**)	Bohai Sea	*Penicillium* sp. XL-01	HeLa, MDAMB-231, MGC-803	0.96–3.60 µM	[61]
Norverrucosidinol acetate (**32**)			NRK 52 E	18.3 µM	
Deoxyverrucosidin (**33**)			HeLa, MDAMB-231, MGC-803	1.14–6.37 µM	
Verrucosidinol acetate (**34**)			NRK 52 E	25.0 µM	
Verrucosidin (**35**)			HeLa, MDA-MB-231, MCF-7, MGC-803, A-549, NRK 52 E	1.91–3.91 and 23.1 µM	
2-hydroxyl-3-pyrenocine-thio propanoic acid (**36**)	Marine sediments	*Penicillum citreonigrum* XT20-134	Bel 7402, HT 1080	7.63, 10.22 µM	[62]
Oxalihexane A (**37**)	Red algae *Rhodomela confervoides*	*Penicillium oxalicum* 2021CDF-3	PATU 8988T	93% (20 µM)	[63]
Penidifarnesylin A (**38**)	Marine mud	*Penicillium* sp. ZZ1750	U87MG, U251	5.9, 27.6 µM	[64]
Penifellutins A (**39**)	Deep-sea sediment	*Penicillium crustosum* PR B-2, *Penicillium fellutanum* HDN 14 -323	/	Suppressed zebrafish larval liver proliferation at 10 µM	[65]
Penifellutin B (**40**)					
Radiclonic acid (**41**)	Deep-sea sediment	*Penicillium chrysogenum*	ECA 109, BIU-87, BEL-7402	7.70–13.75 µM	[60]
Penicinones A (**42**)	Rhizospheric soil of mangrove ecosystem	*Penicillium* sp. LA 032	HepG 2, MCF-7, B16	3.87, 30.01, 27.91 µM	[66]
Purpurogenic acid (**43**)	/	*Penicillium purpurogenum* G59	K562, HL-60, HeLa, BGC-823	52.7%, 78.8%, 38.4%, 35.3% (100 μg/mL)	[67]
Penicillic acid (**44**)	Marine sponge	*Penicillium canescens*	L5178 Y	8.9 µM	[48]
Chryxanthone A (**45**)	Red marine alga *Grateloupia turuturu*	*Penicillium chrysogenum* AD-1540	BT-549, HeLa	20.4, 23.5 µM	[68]
Chryxanthone B (**46**)			A549	20.4 µM	
Spenibenzophenones B (**47**)	Mangrove	*Penicillium citrinum* HL-5126	A549	15.7 μg/mL	[69]
3,8-dihydroxy-4-(2,3-dihydroxy-1-hydroxymethylpropyl)-1-methoxYxanthone (**48**)	Deep-sea sediment	*Penicillium chrysogenum*	ECA 109, BIU-87, BEL-7402	>20 and 15.94 µM	[60]
Penicixanthene E (**49**)	Mangrove	*Penicillium* sp. GXIMD 03101	SW 1990	23.8 µM	[70]
4,4′-bond secalonic acid D (4,4′-SAD) (**50**)	Deep-sea sediment	*Penicillium pancreicum*	BGC-823, SGC-7901, HGC-27, EC9706, KYSE450, CNE1, CNE2, SW620, SW480, LOVO, HuH-7, PLC/PRF/5, SK- HEP, Hela, A549, SK- MES-1, SPC-A1, 95D, Jeko-1, Raji, U937, A375, HFF, H22	0.484–1.384 µM	[71]
Averufin (**51**)	Marine sponge	*Penicillium verruculosum* XWSO1F60	HL60	1.005 µM	[73]
Fumigatin chlorohydrin (**52**)	Marine sediments	*Penicillium Brocae* HDN-12- 143	HL-60	18.63 µM	[74]
Iso-fumigatin chlorohydrin (**53**)				24.83 µM	
Endocrocin methyl ester (**54**)	Red snail *Strombus luhuanus* Linnaeus	*Penicillium* sp. WP-13	K562, BEL-7402, SGC-7901	31.49–57.10 µM	[75]
Emodin (**55**)				56.78–87.67 µM	
Emodin (**55**)	Marine sponge	*Penicillium* sp. SCSIO 41015	MGC 803	5.19 µM	[76]
Rugulosin A (**56**)	Sea water	*Penicillium* sp.	QGY7701, H1299, HCT 116	21.2, 18, 17.6 µM	[54]
Penithoketone (**57**)	Deep-sea sediment	*Penicillium thomii* YPGA 3	MCF-7, MDAMB-468, C4-2B, C4-2B/ENZR	4.9–9.1 µM	[77]
3,5-dihydroxy-2-methoxy-1,4-naphthalenedione (**58**)				>50, 38, 41, >50 µM	
2-methoxyjuglone (**59**)				4.9–9.1 µM	
Peniquinone A (**60**)	Rhizosphere soil of *Limonium sinense*	*Penicillium* sp. L129	MCF-7, U87, PC 3	12.39, 9.01, 14.59 µM	[78]
Peniquinone B (**61**)				25.32, 13.45, 19.93 µM	
Embelin A (**62**)	Rhizosphere sediment of mangrove *Aegiceras corniculatum*	*Penicillium* sp. SCSIO 41411	PC-3, LNCaP	18.69, 31.62 µM	[79]
Questiomycin A (**63**)	Marine mud	*Penicillium* sp. ZZ1750	U87 M, U251	14.13–22.56 µM	[80]
β-resoanaparticine A (**64**)	Marine brown alga *Sargassum miyabei*	*Penicillium anacriticum* KMM 4685	LNCaP, DU 145, 22 Rv 1	31–82.5 µM	[85]
8-dehydro-β-resoantarctine A (**65**)					
β-resoanaparticine B (**66**)					
Pensulfonoxy (**67**)	Red alga *Laurencia obtusa*	*Penicillium aculeatum*	HCT-116	5.23 µM	[86]
Pensulfonamide (**68**)			MCF-7, HCT-116	2.18, 6.12 µM	
Peniresorcinoside A (**69**)	Marine mud	*Penicillium* sp. ZZ1750	U87 MG, U251	4.0, 14.1 µM	[64]
Peniresorcinoside B (**70**)				5.6, 9.8 µM	
Peniresorcinoside C (**71**)			U87MG	53 µM	
Peniresorcinoside D (**72**)				19.4 µM	
Peniresorcinoside E (**73**)				22.1 µM	
Sorbicillin (**74**)	Sea water	*Penicillium allii-sativi*	HT-29	5 µM	[87]
Resorcinosides A (**75**)	Deep-sea sediment	*Penicillium janthinellum* 168CLC-17	NUGC-3	9.3 µM	[88]
3,3’-dihydroxy-5,5’-dimethyldiphenyl ether (**76**)	Deep-sea sediment	*Penicillium chrysogenum*	ECA 109, BEL-7402, BIU-87	>20 and 16.41 µM	[60]
Violaceol-II (**77**)			BIU-87, BEL-7402, ECA 109	>20 and 8.95 µM	
Dicitrinone F (**78**)	Marine sediment	*Penicillium citrinum* VM6	A549, MCF 7, MDA-MB-231, Hela, AGS	6.7–29.6 µg/mL	[89]
Phenol acid (**79**)			MCF 7	98.1 µg/mL	
Spirolaxine (**80**)	Arthropod *(Dardanus scutellatus)*	*Penicillium* sp. ZYX-Z-143	BEL-7402	34.35 µM	[90]
Xanthocillin X (**81**)	Marine mud	*Penicillium* sp. ZZ1750	U87 M, U251	13.65–16.33 µM	[80]
(Z)-N-(4-hydroxystyryl)formamide (**82**)	Marine sediments	*Penicillium* sp. SY2107	U251, U87 MG	17.0, 39.8 µM	[91]
Halociline (**83**)	/	*Penicillium griseofulvum*.	SGC-7901, HeLa	0.870, 1.442 µM	[92]
(2’R)-westerdijkin A (**84**)	Marine red alga	*Penicillium chrysogenum* LD-201810	HepG 2	22.0 µM	[93]
(S)-(+)-11-dehydrosydonic acid (**85**)			A549, THP-1	21.2, 18.2 µM	
Penicacid F (**86**)	Marine sediments	*Penicillium parvum* HDN17-478	HCT-116, BEL-7402, MGC-803, SH-SY 5 Y, HO-8910, HL-60	12.61–26.38 µM	[94]
Penicacid G (**87**)					
Mycophenolic acid (**88**)				1.69–12.98 µM	
Mycophenolic methyl ester (**89**)					
2-(2-aminopropanamido)benzoic acid (**90**)	Soft coral	*Penicillium chrysogenum VH 17*	MCF 7, HepG 2	87.17, 97.32 µM	[95]
Dehydrocurvularin (**91**)	Marine mangrove *Bruguiera sexangular*	*Penicillium sumatrense* MA-325	HCT 116, 786-O, 5673, Hela	3.5–14.9 µM	[96]
5,5-dichloro-1-(3,5-dimethoxyphenyl)-1,4-dihydroxypentan-2-one (**92**)	Marine sediments	*Penicillum citreonigrum* XT20-134	Bel 7402, HT 1080	13.14, 16.53 µM	[62]
Sorbicillfuran B (**93**)	Marine alga *Coelarthorum* sp.	*Penicillium citrinum* SCSIO 41402	HL-60	9.6 µM	[97]
Sorbicatechol D (**94**)	Sea water	*Penicillium allii-sativi*	HT-29	30 µM	[87]
Penipyridinone B (**95**)	Marine mud	*Penicillium* sp. ZZ1750	U87 M, U251	2.45, 11.40 µM	[80]
Terrein (**96**)	Marine sediments	*Penicillium citreonigrum*	HeLa	11.3 µM	[59]
LAMA (**97**)	Marine mud	*Penicillium chrysogenum* strain S003	A-549, HeLa, DU-145, HepG2, MCF-7	>100 µM	[56]
Meleagrin (**98**)	Marine sediments	*Penicillium* sp. YPGA11	EC109, EC9706, KYSE70, KYSE450, HepG2	25.03–36.93 and 7.0 µM	[101]
Glandicoline B (**99**)			EC109, EC9706, KYSE70, KYSE450	30.11–55.379 μM	
Deoxy-14,15-dehydroisoaustamide (**100**)	Soft coral	*Penicillium dimorphosporum* KMM 4689	/	Enhancing AR-targeted therapy efficacy	[105]
Peniokaramine (**101**)	Marine sediments	*Penicillium* sp. LSH-3-1	A549	53.43% (50 μM)	[106]
Penicindopene A (**102**)	Deep-sea water	*Penicillium* sp. YPCMAC1	A549, HeLa	15.2, 20.5 µM	[107]
Emindole SB (**103**)	Bivalve mollusk (*Meretrix lusoria*)	*Penicillium* sp. KFD 28	K562	18.8 µM	[108]
Spirotryprostatin G (**104**)	Bohai Sea	*Penicillium brasilianum* HBU-136	HL-60	6 µM	[109]
Cyclotryprostatin F (**105**)			MCF-7	7.6 µM	
Cyclotryprostatin G (**106**)			MCF-7	10.8 µM	
Spirocyclic diketopiperazine alkaloid (**107**)			HL-60	7.9 µM	
Cyclotryprostatin B (**108**)			MCF-7	5.1 µM	
Roquefortine J (**109**)	Marine sediments	*Penicillium granulatum*	HepG3	19.5 µM	[102]
Asperinamide B (**110**)	Marine red alga	*Penicillium pesticum* 2021 CDF-3	FADU	0.43 µM	[110]
Peniochroloid A (**111**)			A549	29.84 µM	
Peniochroloid B (**112**)			A549	15.30 µM	
Penicimutanin C (**113**)	Bohai Sea	*Penicillium purpurogenum* G59	K562, HL-60, HeLa, BGC-823, MCF-7	11.9, 5.0, 8.6, 8.7,6.0 µM 10.7, 6.1, 7.0, 8.3, 7.3 µM	[111]
Penicimutanin A (**114**)					
Viridicatol (**115**)	Marine mud	*Penicillium solitum*	PANC-1, Hela, A549	18, 19, 24 µM	[112]
Perpyrrospirone A (**116**)	/	*Penicillium citrinum* DY180712	MGC 803, HepG 2, MDA-MB-231, MCF-7, Bel-7402, HeLa	2.5–38.9 µM	[113]
Penicillione G (**117**)					
Penicipyrroether A (**118**)	Marine crab *Pachygrapsus crassipes*	*Penicillium* sp. ZZ380	U87 MG, U251	1.64, 5.50 µM	[114]
Pyrrospirone J (**119**)				10.52, 17.92 µM	
Pyrrospirone G (**120**)	Marine crab *Pachygrapsus crassipes*	*Penicillium* sp. ZZ380	U87MG, U251, SHG44, C6	1.06–8.52 µM	[115]
Pyrrospirone H (**121**)				7.44–26.64 µM	
Pyrrospirone I (**122**)					
Polonimide A (**123**)	Bohai Sea	*Penicillium polonicum* MN 623481	A549, HGC-27, UMUC-3	>10 µM	[116]
Polonimide B (**124**)					
Polonimide C (**125**)					
Aurantiomide C (**126**)					
Anacine (**127**)				6.0, 6.2, 7.2 µM	
Aurantiomide A (**128**)				>10 µM	
Aurantiomide B (**129**)					
Cyclopenol (**130**)	Marine sediments	*Penicillium chrysogenum*	BIU-87, BEL-7402	8.34, 7.81 µM	[60]
Compound (**131**)	Marine sponge *Petrosia* sp.	*Penicillium citrinum* 2015 PF 07	HCT 115, MCF-7	20 μg/mL	[117]
Compound (**132**)					
Penicillatide B (**133**)	Red Sea tunicate *Didemnum* sp.	*Penicillium* sp.	HCT-116	6 µM	[118]
Cyclo(R-Pro–S-Phe) (**134**)				9.57 µM	
Adametizine C (**135**)	Mangrove sediment	*Penicillium ludwiglium* SCSIO 41408	22 Rv 1, PC-3	13.9, 44.0 µM	[119]
Adametizine A (**136**)			22 Rv 1	13.0 µM	
DC1149B (**137**)			22 Rv 1, PC-3	13.6, 5.1 µM	
Inonotic acid C (**138**)	Marine alga	*Penicillium puericum* MZY-202312-521	MCF-7	7.7 µM	[123]
Purpuride G (**139**)	Deep-sea sediment	*Penicillium minioluteum* ZZ 1657	U251, U87 MG	4.49, 10.9 µM	[124]
Decumbenone A (**140**)	Marine sediments	*Penicillium chrysogenum*	ECA 109	12.41 µM	[60]
Decumbenone B (**141**)				15.60 µM	
1-acetylpallidopenilline A (**142**)	Marine grass	*Penicillium yezoense* KMM 4679	MCF-7	0.66 µM	[125]
Copteremophilane D (**143**)	Sponge of *Xestospongia testudinaria*	*Penicillium copticola* WZXY-m122-9	HCT-8	5.4 µM	[126]
Copteremophilane E (**144**)			HCT-8	7.3 µM	
Copteremophilane G (**145**)			A549	>10 µM	
Copteremophilane H (**146**)			A549	3.23 µM	
Meroanapartine A (**147**)	Marine Brown alga *Sargassum miyabei*	*Penicillium anacuticum* KMM 4685	/	P-gp-inhibitory activity	[127]
Meroanapartine B (**148**)					
Meroanapartine C (**149**)					
Penicimeroterpenoid A (**150**)	Soft coral	*Penicillium* sp. SCSIO 41512	CDC 25 B	20 µM	[128]
Penicimeroterpenoid B (**151**)					
Penicimeroterpenoid C (**152**)					
Penimeroterpenoid A (**153**)	Marine sediments	*Penicillium* sp. A18	A549, HCT 116, SW 480	82.61, 78.63, 95.54 µM	[129]
Penisimplinoid F (**154**)	Sponge	*Penicillium simplicissimum* 19 XS 15 ZM-3	NCI-H446	6.49 µM	[130]
Andrastone A (**155**)	Deep-sea water	*Penicilliumallii-sativi*	HepG 2	7.8 µM	[131]
Austalide Y (**156**)	Sea water	*Penicillium thomii* YPGA 3	MDA-MB-468	38.9 μM	[132]
Stachybotrylactone B (**157**)	Soft coral	*Penicillium* sp. SCSIO 41201	HL-60, K562, MOLT-4, ACHN, 786-O, OS-RC-2	4.12–23.55 µM	[133]
Breviones I (**158**)	Soft coral	*Penicillium* sp. TJ403 -1	HL-60, A-549, HEP 3B	4.92, 8.60, 5.50 µM	[134,135]
Diaporthein B (**159**)	Intestinal tract of Onchidium struma	*Penicillium sclerotiorum* GZU-XW 03 -2	HCT 116, LOVO	1.5, 3 µM	[136]
Conidiogenol D (**160**)	Marine sediments	*Penicillium* sp. SY2107	EC109, EC9706, KYSE30, KYSE70, KYSE450	36.80–54.7 µM	[101]
Conidiogenone C (**161**)				27.05–42.13 µM	
22-triene-3,5-diol (**162**)	Marine grass	*Penicillium leveling* N33.2	Hep-G2, A549, MCF-7	2.89–18.51 µg/mL	[137]
Solitumergosterol A (**163**)	Marine sediments	*Penicillium solarum*	MB231	44.1% (20 μM)	[138]
Ergosterol (**164**)	Marine sediments	*Penicillium chrysogenum* strain S003	A-549, DU-14, MCF-7, HepG 2	21.26, 1.50, 16.95, 2.89 µM	[56]
Epidioxyergosterol (**165**)				19.3, 6.10, 13.6, 3.07 µM	
16a-methylpregna-17a,19-dihydroxy-(9,11)-epoxy-4-ene-3,18-dione-20-acetoxy (**166**)	Sponge	*Penicillium citrinum* SCSIO 41017	SF-268, MCF-7, HepG-2, A549	13.5–18.0 µM	[51]
Penicisteroid E (**167**)	Marine sediments	*Penicillium granulatum*	A549, BIU-87, BEL-7402, ECA-109, Hela-S3, PANC-1	14.4–4.1 µM	[139]
Penicisteroid G (**168**)					
Penicisteroid H (**169**)					
Penicisteroid A (**170**)					
Penicisteroid C (**171**)					
Isonuatigenin I (**172**)	Marine sediments	*Penicillium granulatum*	HepG2	8.6 µM	[102]
Penicisteroid A (**173**)				8.2 µM	
5 α, 6 α-epoxy-(22 E, 24 R)-ergosta-8(14), 22-diene-3 β,7 α-diol (**174**)	Soft coral	*Penicillium chrysogenum* VH17	HepG2, A549, MCF 7	29.43, 33.02, 36.72 µM	[95]

## Data Availability

No new data were created or analyzed in this study. Data sharing is not applicable to this article.
